# Targeting the cancer cells and cancer‐associated fibroblasts with next‐generation FGFR inhibitors in prostate cancer co‐culture models

**DOI:** 10.1002/cam4.70240

**Published:** 2024-09-20

**Authors:** Syeda Afshan, Yu Gang Kim, Jesse Mattsson, Malin Åkerfelt, Pirkko Härkönen, Martin Baumgartner, Matthias Nees

**Affiliations:** ^1^ FICAN West Cancer Centre Institute of Biomedicine, University of Turku Turku Finland; ^2^ Cell Biology, Faculty of Science and Engineering Åbo Akademi University Turku Finland; ^3^ Pediatric Molecular Neuro‐Oncology Research Laboratory University Children's Hospital Zurich Zurich Switzerland; ^4^ Department of Biochemistry and Molecular Biology Medical University of Lublin Lublin Poland; ^5^ Present address: Korea Mouse Phenotyping Center (KMPC) Seoul National University Seoul South Korea; ^6^ Present address: DelSiTech Ltd Turku Finland

**Keywords:** AR antagonist, cancer‐associated fibroblasts, castration resistant prostate cancer, darolutamide, enzalutamide, FGFR inhibitors, FRS2α inhibitor

## Abstract

**Background:**

Inhibition of androgen receptor (AR) signaling is the main treatment strategy in advanced prostate cancer (PCa). A subset of castration resistant prostate cancer (CRPC) bypasses the AR blockade by increased fibroblast growth factor receptor (FGFR) signaling. The first‐ and second‐generation, non‐covalent FGFR inhibitors (FGFRis) have largely failed in the clinical trials against PCa. **Purpose:** In this study, we tested the drug sensitivity of LNCaP, VCaP, and CWR‐R1PCa cell lines to second‐generation, covalent FGFRis (FIIN1, FIIN2) and a novel FGFR downstream molecule inhibitor (FRS2αi).

**Methods:**

2D and 3D mono‐ and co‐cultures of cancer cells, and cancer‐associated fibroblasts (CAFs) were used to mimic tumor‐stroma interactions in the extracellular matrix (ECM). The treatment responses of the FGFR signaling molecules, the viability and proliferation of cancer cells, and CAFs were determined through immunoblotting, migration assay, cell viability assay, and real‐time imaging. Immunofluorescent and confocal microscopy images of control and treated cultures of cancer cells and CAFs, and their morphometric data were deduced.

**Results:**

The FGFRis were more effective in mono‐cultures of the cancer cells compared with co‐cultures with CAFs. The FRS2αi was specifically effective in co‐cultures with CAFs but was not cytotoxic to CAF mono‐cultures as in the case of FIIN1 and FIIN2. At the molecular level, FRS2αi decreased p‐FRS2α, p‐ERK1/2, and activated apoptosis as monitored by cleaved caspase‐3 activity in a concentration‐dependent manner in the co‐cultures. We observed no synergistic drug efficacy in the combination treatment of the FGFRi with ARi, enzalutamide, and darolutamide. The FRS2αi treatment led to a decrease in proliferation of cancer cell clusters in co‐cultures as indicated by their reduced size and Ki67 expression.

**Conclusions:**

CAFs exert a protective effect on cancer cells and should be included in the in vitro models to make them physiologically more relevant in screening and testing of FGFRis. The FRS2αi was the most potent agent in reducing the viability and proliferation of the 3D organotypic co‐cultures, mainly by disrupting the contact between CAFs and cancer cell clusters. The next‐generation FGFRi, FRS2αi, may be a better alternative treatment option for overcoming ARi treatment resistance in advanced PCa.

## Graphical abstract

Physiologically relevant contact and possible interactions between the cancer cells and cancer‐associated fibroblasts (CAFs) when closely replicated in the *in vitro* co‐cultures, decrease the effect of the FGFR inhibitors (FGFRis) compared to their respective mono‐cultures. Targeting the FGFR‐specific adaptor protein, FRS2 using a novel FRS2ai has shown to have a specific and anti‐ proliferative effect by disrupting the contact between the CAFs and cancer cells.

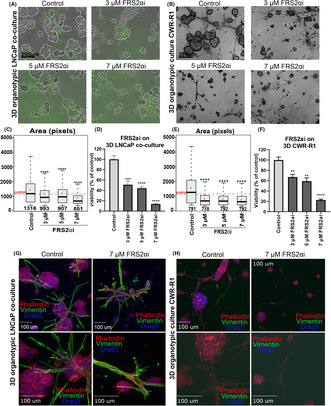

## 1 INTRODUCTION

Prostate cancer (PCa) is primarily regulated by androgens via the androgen receptor (AR)^1^ and further stimulated by modulation of various signaling pathways including fibroblast growth factor receptor (FGFR) signaling. Localized PCa is routinely treated by radical prostatectomy and/or radiation therapy. In cases where PCa has advanced despite prostatectomy or PCa is primarily metastasized, the patient is typically treated with androgen deprivation therapy (ADT), in which androgen effects are suppressed by inhibiting androgen synthesis indirectly or directly,^2^ or by blocking their activity using AR inhibitors (ARis) such as enzalutamide and darolutamide.^3^ However, 10%–20% of PCa patients develop resistance to ADT and the tumor progresses to castration‐resistant PCa (CRPC).^4^, ^5^ Therefore, it is important to identify alternative treatment strategies specifically targeting such advanced PCa, and/or to overcome the PCa resistance to ADT. One of the molecular mechanisms frequently observed in ARi‐resistant cells is the activation of FGFR signaling.^6^, ^7^

Fibroblast growth factors (FGFs) and their five receptors (FGFR 1–4 and FGFRL1) are involved in the differentiation, migration, and proliferation of cancer cells.^8^, ^9^ The FGFRs (except FGFRL1) have an intracellular tyrosine kinase binding domain, which is activated after binding to ligands such as FGF2. This leads to phosphorylation and activation of the key downstream FGFR mediator p‐FRS2α, which in turn activates the intracellular cell cycle cascade ERK1/2, PI3Kinase/AKT, and PLCγ/MAPK signaling leading to cancer progression including in PCa.^10^, ^11^, ^12^ Mutations, overexpression, deletions, and gene fusions involving FGFRs, or functional deregulation of their downstream signaling pathways have been widely associated with many cancers incuding PCa.^13^, ^14^, ^15^ FGFR signaling depends on ligands secreted in a paracrine, juxtacrine, and/or autocrine fashion by the cancer cells themselves but typically also by cancer‐associated fibroblasts (CAFs) or other stromal cells found in solid tumor tissues.^16^, ^17^

Detailed studies based on single‐cell RNA sequencing have shown that the response to drug treatments in PCa is affected by the properties of both the epithelium‐derived tumor cells and their interactions with the tumor microenvironment (TME), which includes CAFs and the extracellular matrix (ECM).^18^ CAFs secrete growth factors, cytokines, exosomes or extracellular vesicles (EVs), and ECM proteins,^19^ and represent a consistent although highly diverse component of the TME in PCa tissues.^20^, ^21^ Specifically, different subtypes of CAFs have been recently identified, which protect and support tumor cells, or interact with infiltrating immune cells and other cellular components of the TME.^22^, ^23^ Moreover, the traditional monolayer or two‐dimensional (2D) in vitro cell cultures on plastic surfaces do not adequately capture the complex morphological, cellular, and phenotypic heterogeneities of PCa tissues.^24^ For that reason, several three‐dimensional (3D), or organotypic cell culture techniques have been developed over the past decade, which claim to represent physiologically more relevant model systems that may mimic the initiation and progression of human cancers including prostate cancers.^25^, ^26^ These complex models include organotypic 3D cultures, typically embedded in ECM preparations or hydrogels such as Matrigel and/or collagen type I, and are increasingly used for in vitro chemosensitivity testing and personalized medicine applications. These hydrogels support cells to actively engage in forming cell–cell and cell–matrix interactions that at least partly mimic the dynamics and the architecture of in vivo tumor tissues.^27^, ^28^ For this study, representative PCa cell lines were chosen based a differential response to androgen signaling, as observed in the clinical progression of PCa. This response is ranging from AR‐dependent (VCaP),^29^ to androgen‐sensitive (LNCaP),^30^ and finally, largely androgen‐independent (CWR‐R1).^31^ The CWR‐R1 cells are dependent on the persistent, growth‐stimulating nature of mouse CAFs that persist in this line and originate from the patient‐derived xenografts (CWR22 model) used to establish this model.^32^, ^33^ We developed reproducible, standardized co‐cultures of tumor cells from PCa cell lines (VCaP and LNCaP) with patient‐derived immortalized CAFs,^28^, ^34^, ^35^ followed by automated analysis of confocal microscopy images^34^, ^36^ as a phenotypic or high‐content readout. The human telomerase reverse transcriptase (hTERT) expression in these immortalized CAFs showed only mild directional effects interpreted from a global expression profiling analysis using cDNA microarrays on RNA samples from immortalized CAFs versus non‐immortalized CAFs.^37^

The above mentioned co‐culture models of cancer cells and CAFs were used to test our hypothesis that advanced PCa may benefit from the treatment with next‐generation FGFRi alone and/or in combination with ARi. The next‐generation FGFRi includes the second‐generation, covalent‐binding, irreversible FGFRi such as FIIN1 and FIIN2,^38^, ^39^, ^40^ which bind to the kinase domain of the FGFRs have shown to overcome the resistance of first‐generation FGFRi due to gatekeeper mutations of FGFRs.^41^ In addition, we tested a novel concept of targeting fibroblast growth factor receptor substrate 2 (FRS2*α*), which integrates FGFR signaling from all four FGFR receptors in normal and cancer cells. Specifically, this exploits the critical downstream signaling molecule FRS2α,^42^, ^43^ which is phosphorylated upon binding of ligand to any of the four active FGFRs. FRS2α is specifically and effectively blocked by a recently developed, novel experimental inhibitor (“compound 7”)^44^ which is used in this study.

## 2 MATERIALS AND METHODS

### 2.1 Cell culture

Human PCa cell lines LNCaP (*RRID: CVCL_1379*, clone FGC), CWR‐R1 (*RRID: CVCL_4833*), and VCaP (*RRID: CVCL_2235*) were obtained from the American Type Culture Collection (ATCC). The immortalized PF179T CAFs (*RRID: CVCL_JL59*) isolated from a prostate cancer biopsy was obtained from Varda Rotter, Weizmann Institute, Israel.^37^ CWR‐R1, VCaP, and GFP‐labeled PF179T CAF were cultured in DMEM containing Glutamine, 10% fetal bovine serum (FBS, Gibco, Thermo Fischer, UK), and 1% penicillin/streptomycin (Lonza, Belgium). LNCaP cells were cultured in RPMI medium supplemented with 10% FBS, 2 mM UltraGlutamine (Lonza, Belgium), and 1% penicillin/streptomycin (Lonza, Belgium). All cell lines were maintained at 37°C with 5% CO_2_. The cell lines have been authenticated using short tandem repeat profiling at IdentiCell Laboratories, Department of Molecular Medicine, Aarhus University Hospital, Denmark, and at the Institute of Molecular Medicine (FIMM), University of Helsinki, Finland. All cells were free of mycoplasma (routinely tested with Lonza LT07‐118 mycoplasma detection kit).

### 2.2 Quantitative reverse transcriptase‐polymerase chain reaction (qRT‐PCR)

The total RNA was extracted from cells in culture using the RNeasy Mini Kit (Qiagen, Germany). 1 μg of extracted RNA was transcribed into complementary DNA using a master mix of RNase inhibitor (Promega, USA), Oligo dT primer (Oligomer Oy, Finland), dNTP mix (Thermo Fisher Scientific, Finland), and Maxima reverse transcriptase (Thermo Fisher Scientific, Finland). The qRT‐PCR was carried out using SYBR™ Green master mix (Thermo Scientific, Finland) with a Bio‐Rad CFX384 Touch™ system. The primers used for FGFR1 (F: 5′TGGCACCCGAGGCATTATTT3′, R: 5′CATGTACAGCTGGTTGTTGC3′), FGFR2 (F: 5′AACAGTCATCCTGTGCCGAA3′, R: 5′AGCCGAAACTGTTACCTGTC3′), FGFR3 (F: 5′CGTCCA CCGACGAGTACCT3′, R: 5′ CTCACATTGTTGGGGACCAGT3′), FGFR4 (F: 5′CTGACACAGTGCTCGACCTT3′, R: 5′ AACCCTGACATTTGGGCCAT3′) were designed using the NCBI databases (http://www.ncbi.nlm.nih.gov/tools/primer‐blast/).^45^ The standard ΔΔCt method was used to calculate fold‐change differences in the mRNA expression. They were normalized to the housekeeping gene, TATA box binding protein (TBP) (F:5′GAATATCCCAAGCGGTTT3′, R:5′ ACTTCACATCACAGCTCCCC3′).

### 2.3 2D cell proliferation and viability assay after inhibitor treatment

FGFRi, FIIN1, and FIIN2 were obtained from SelleckChem, and FRS2α inhibitor (“compound C7”)^44^ was obtained from Dr. Martin Baumgartner, University of Zurich, Switzerland. Compound 7 is referred to as FRS2αi in this study. ARi, enzalutamide (MDV3100) and darolutamide (ODM‐201) were purchased from AdooQ Biosciences. These inhibitors were dissolved in Dimethyl Sulfoxide (DMSO, Fischer Scientific, USA), and corresponding DMSO concentrations (0.1%–0.2% DMSO) were used in control samples based on single or combination treatments. The biochemical IC50 values of the inhibitors are reported in Table S1. LNCaP, VCaP cells in 2D mono‐culture and 2D co‐culture with PF179T CAFs (ratio of 5 cancer cells:1 CAFs), and CWR‐R1 were seeded in 96‐well plates in their respective growth medium for 24 h before drug treatment. The final treatment media contained 0.3 μM–10 μM inhibitors in 5% FBS and supplemented with 25 ng/mL FGF2 and 0.1 nM—1 nM synthetic androgen (R1881, Sigma, Germany). The cells were imaged every 6 h with Incucyte® S3 live cell imaging (Sartorius Stedim Biotech, France). The proliferation was monitored for 72 h after drug treatment and kinetic data from phase‐contrast images and fluorescent CAFs were analyzed with Incucyte® S3 software. Cell Titre Glo 2.0 (Promega, USA) was used to measure the effect of inhibitor treatment on cellular viability after 72 h. The data representation was plotted using GraphPad Prism 8 software.

### 2.4 Immunoblotting

In case of FGFRi treatment, cells were serum starved for 24 h–36 h in DMEM or RPMI with 1% Bovine Serum Albumin (BSA, Thermo Fischer, Finland) before treatment with FGFRi for 30 min (FIIN1 or FIIN2), 16 h (FRS2αi) and 25 ng/mL of FGF2 (R&D system, Canada) with 4 μg/mL Heparin (ScienCell research laboratories, USA) for 15 min before collection of cell lysates. In case of ARi, androgen‐deprived cells in charcoal‐stripped serum (Gibco, Thermo Fischer, UK) for 48 h were exposed to enzalutamide and darolutamide, and synthetic androgen (R1881) for 72 h. Cell lysates were collected in cell lysis buffer supplemented with a protease inhibitor and phosphatase inhibitor cocktail (Pierce Ltd, Thermo Fischer Scientific, USA). The extracted protein was combined with Laemmli buffer (Bio‐rad, Finland) containing β‐mercaptoethanol, denatured by boiling, and separated on 4–20% Mini‐PROTEAN® TGX™ precast gradient protein gels (Bio‐Rad, Finland), and transferred for 2 h at 180 V using Towbin transfer buffer (Biorad, Finland) or for 30 min using the semidry gel transfer system (Bio‐Rad, Finland) and Trans‐Blot Turbo Midi 0.2 μm Nitrocellulose Transfer Packs (Bio‐Rad, Finland). After overnight incubation with primary antibodies diluted in 5% BSA [1:1000 PSA #ab53774, 1: 400 AR #MS‐441‐P1, 1:1000 CAMKK2 #HPA014659, 1:1000 p‐FRS2α (Y196) #CST 3864, 1:1000 p‐FRS2α (Y436) #CST 3861, 1:1000 pERK1/2 #CST 9102, 1:1000 ERK1/2 #CST 9101, 1:500 FRS2α(H‐91) #sc8318, 1:1000 α‐tubulin #ab4074] at 4°C, membranes were incubated with secondary antibodies labeled with fluorescent dyes IRDye® 800CW/600CW donkey anti‐rabbit IgG or donkey anti‐mouse IgG (LI‐COR, USA) for 1 h at room temperature. The signals were visualized in Odyssey® CLx Imaging System (LI‐COR, USA).

### 2.5 Cell migration assay

Cell migration was examined using scratch wound assay in Incucyte® S3 imaging device (Sartorius Stedim Biotech, France). Briefly, the 96 well plates were seeded with CWR‐R1 cells and incubated in standard conditions for 24 h–48 h to reach 100% confluence. The scratch wounds were made in confluent cell layers using the wound‐maker tool. The inhibitors in the required concentrations (0.3 μM–10 μM) were added on top of the wounded cell layer. The percentage of cell migration leading to relative wound density was analyzed with Incucyte® S3 software.

### 2.6 3D organotypic cultures and inhibitor treatments

3D organotypic mono‐cultures and co‐cultures were prepared in Matrigel (Corning/BD Biosciences, USA) and Collagen type 1 (BD Biosciences, USA) matrices as described previously.^46^ LNCaP cells with PF179T CAFs (5:4) in 2:1 mix of Matrigel and Collagen type 1, and CWR‐R1 in 1:1 mix of the matrices were seeded in their respective growth media on 96‐well angiogenesis μ‐plates (Ibidi, Germany). The single cells replicate and form organotypic cultures within 4 days and were treated with inhibitors for an additional period of 6 days with a media change every 2 days. The single treatment with FIIN1, FIIN2, and FRS2αi were performed for a range of concentrations between 0.3 μM and 10 μM and combination treatments with 1 μM enzalutamide or darolutamide. The effects of inhibitor treatment on cell viability were measured by spectrophotometry using Cell Titre Glo 2.0 (Promega, USA).

### 2.7 3D organotypic culture live cell staining and imaging

The 3D organotypic cultures were monitored once a day using the Incucyte® S3 imaging device. At the end of inhibitor treatments on day 10 after cell seeding, organotypic structures were stained with 1 μg/mL of Calcein AM (488 nm) (Thermo Fisher Scientific, MA, USA) to visualize the live cells. The resulting stained cultures were imaged with a spinning disk confocal microscope (3i CSU‐W1, Zeiss 5x objective, Germany). Maximum intensity projections and batch normalization of image stacks were done with SlideBook6 software (3I Inc., CO, USA). The organotypic cultures in the images were subjected to segmentation and then analyzed using AMIDA software.^34^, ^47^ The resulting quantitative morphometric data such as *area* (in pixels), symmetry or *roundness*, and features typical of cell invasion such as *appendages* of the segmented organoid were plotted using the R software (http://www.r‐project.org).

### 2.8 Immunofluorescence staining

The 3D organotypic co‐cultures were fixed with 4% paraformaldehyde. Cells were permeabilized with 0.5% Triton X‐100 and blocked with 3% BSA. The fixed cultures were then washed, and incubated overnight at 4°C with primary antibodies (rabbit Cytokeratin 8+18/1:300/Abcam #53280, UK; mouse α‐actin/1:200/Santa Cruz #32251, USA; mouse Ki67/1:100/Dako #M7240, USA; rabbit Ki67/1:100/Abcam #ab15580, UK; rabbit Vimentin/1:200/cell signaling technology #5741 for mouse CAFs and Abcam #ab18200 antibody for human CAFs), washed with PBS, and finally incubated at room temperature for 1 h with secondary antibodies (goat anti‐mouse and anti‐rabbit IgG‐Alexa Flour Plus 488 and 555/1:200; Thermo Fischer Scientific, USA). Phalloidin Alexa Flour 555 to stain actin filaments and Draq5 (647 nm/1:1000, Thermo Fischer Scientific, USA) was used as DNA counterstain. The images were recorded with a 3i CSU‐W1 spinning disk confocal microscope using Zeiss 20x/0.8 Plan‐Apochromat and 40x/0.6 LD Plan‐Neofluar objectives (3I GmbH, Germany).

### 2.9 Statistical analysis

GraphPad Prism 8 and R software (http://www.r‐project.org) were used for visualization of data and statistical analyses. The statistical methods used are mentioned in the captions under each figure.

## 3 RESULTS

### 3.1 Expression and activation of FGFRs in PCa cell lines

The PCa cell lines LNCaP, VCaP, CWR‐R1, and the PF179T CAFs all express FGFR 1–4. The mRNA expression profile of the cell lines shows some similarities, with FGFR2 and FGFR3 being typically expressed at higher levels compared to FGFR1, but the cell lines differ in terms of FGFR4 expression. CWR‐R1 cells, which maintain a stable population of (mouse) fibroblasts, expressed significantly higher levels of all FGFRs, compared to LNCaP and VCaP. In contrast, PF179T CAFs express mainly FGFR1 and show only low levels of all other FGFRs (Figure S1).

### 3.2 Second‐generation FGFR inhibitors (FIIN1, FIIN2) block FGFR‐mediated signaling pathway in PCa cell lines but cause marked cytotoxic off‐target effects

**FIGURE 1:**
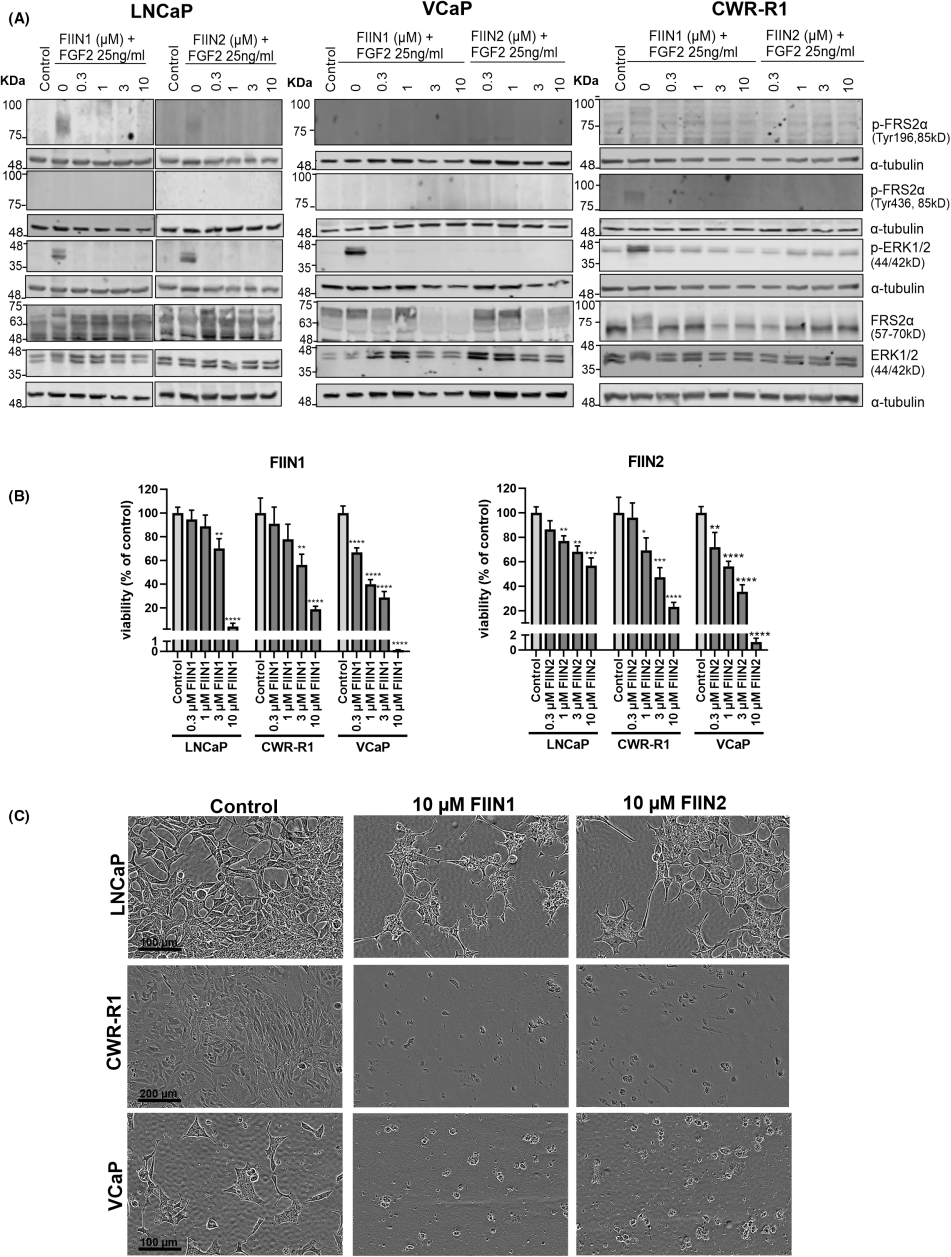
The effects of second‐generation pan‐FGFRi, FIIN1, and FIIN2 on FGFR signaling in PCa cells. (A) Immunoblot analysis of FGFR pathway activation of serum‐starved LNCaP, VCaP, and CWR‐R1 cells treated with FGF2 for 15 min in the presence or absence of FIIN1 and FIIN2 at indicated concentrations and control sample being 0.1% DMSO. Total FRS2α and ERK1/2 protein were used as controls for phospho‐specific antibodies, and α‐tubulin as a loading control. (B) A biochemical end‐point assay (Cell Titre Glo 2.0) to determine the dose–response of treatment effects. Statistical significance of *n* = 3 replicas was calculated using one‐way ANOVA, combined with Dunnett's test with untreated control as reference (*p < 0.05, **p < 0.01, ***p < 0.001, ****p < 0.0001). (C) Visualization of treatment effect by phase‐contrast microscopy imaging of treated and its control cultures at the endpoint after 72 h treatment with FIIN1 and FIIN2 inhibitors. The scale bar is as mentioned in the image.

The serum‐starved PCa cell lines, LNCaP, VCaP, and CWR‐R1 were treated with second‐generation FGFR inhibitors (FIIN1, FIIN2) that covalently bind to the receptor, along with the ligand FGF2. The exposure of PCa cell lines to FGF2 showed variable extents of activation, as indicated by phosphorylation of FRSα, and ERK1/2 (Figure 1A). In LNCaP cells, a band of p‐FRS2α at Tyr 196 was observed. This was further reduced after exposure to even the lowest concentrations of FIIN1/2 inhibitors, indicating low‐level activation of FGFR signaling. In contrast, VCaP cells did not show any phosphorylation of FRS2α even after exposure to FGF2. In both LNCaP and VCaP cell lines, phosphorylated ERK1/2 protein could be detected, which was rapidly diminished by FGFRi. Phosphorylation of ERK1/2 in these cell lines may be partly induced by upstream signaling events that are only indirectly linked to FGF‐FGFR signaling. In contrast, the cell line CWR‐R1 showed rapid and consistent phosphorylation of FRS2α at both the Tyr 196 and Tyr 436 sites, and strong phosphorylation of downstream ERK1/2 upon exposure to ligand FGF2. This persistent activation could not be completely abolished even with a high concentration of FGFRis. We conclude that the FGFRis, FIIN1, and FIIN2 inhibit the phosphorylation FRS2α already at the lowest concentration (0.3 μM) tested and partially decrease the phosphorylation of ERK1/2 in CWR‐R1 (Figure 1A). CWR‐R1 cells appear to depend on FGFR signaling for proliferation and are sensitive for growth inhibition by FGFRi. In the viability assay, only at the highest concentration tested (10 μM), FIIN1 and FIIN2 were found to be cytotoxic for all three PCa cell lines (Figure 1B). At lower concentrations, FIIN1 and FIIN2 showed effective growth inhibition of CWR‐R1 cells at approximately 3 μM, while not even a 50% inhibition was achieved at <10 μM in the poorly responsive LNCaP cells. Despite the lack of specific pFRS2α activation, VCaP cells showed even higher sensitivity to treatment than CWR‐R1 cells (approx. 70% inhibition of cell viability at 3 μM) (Figure 1B).

All PCa cell lines showed dose‐dependent growth inhibition, as indicated by a decrease in the total cell number and viability, and also morphometric responses (Figure 1C), essentially confirming the viability assay data. The highest concentrations of FIIN1 and FIIN2 (10 μM) most probably resulted in nonspecific, off‐target effects and were excluded from further analyses.

### 3.3 CAFs in 2D and 3D co‐culture with PCa cells exert a significant protective effect against FGFRi treatment

**FIGURE 2:**
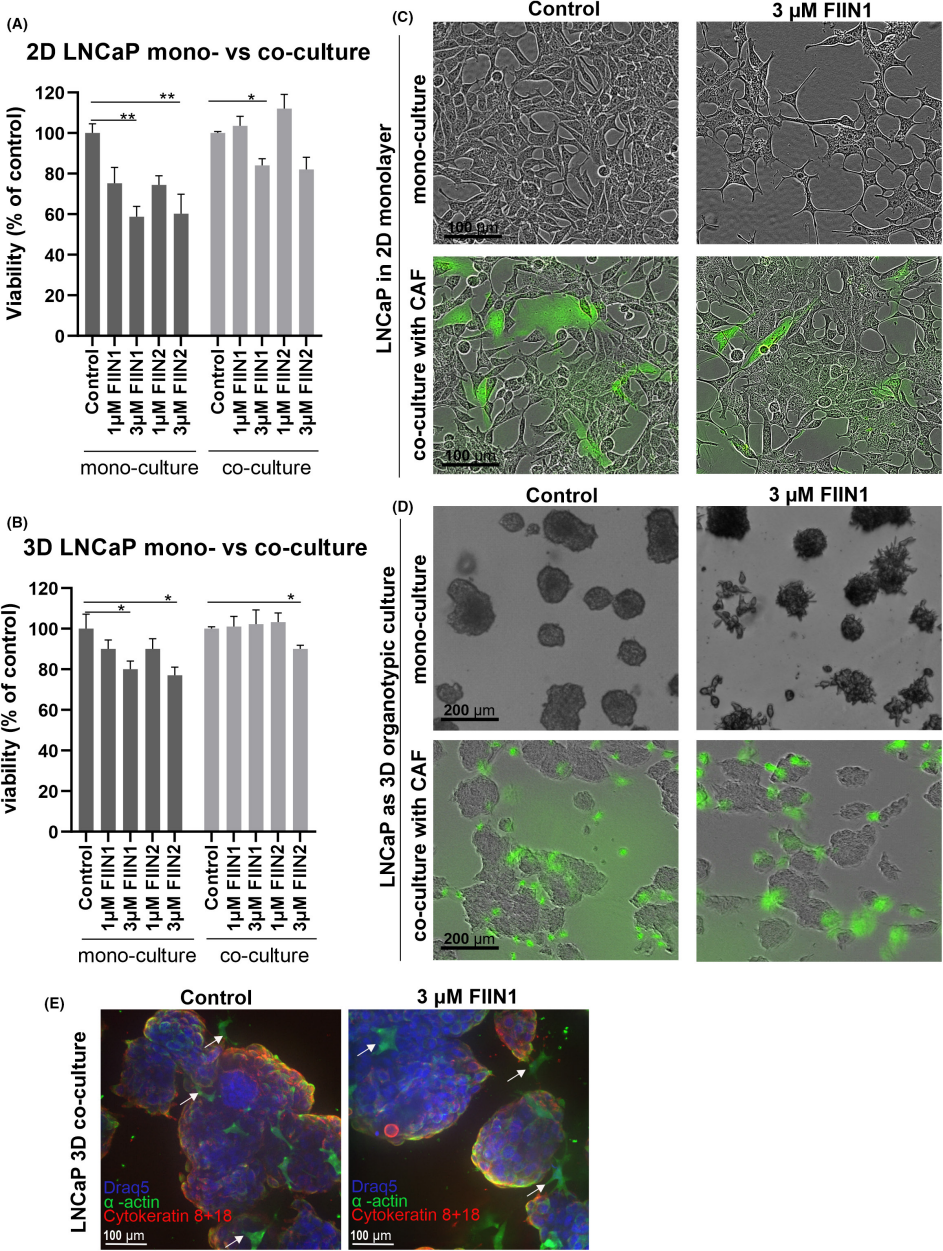
Reduction of cell viability (in percentage, compared to control) and morphologic effects as the result of FGFRi (FIIN1, FIIN2) exposure in adherent 2D monolayer versus matrix‐embedded 3D organotypic cultures with or without CAFs (green fluorescence). The treatment was done for 3 days in 2D cultures and 6 days in 3D cultures in three replicas. (A) The drug sensitivity of LNCaP cells quantitated in 2D mono‐culture and co‐culture with CAFs, as measured by Cell Titre Glo metabolic assay. Similarly, (B) the viability of LNCaP cells in 3D mono‐ versus co‐culture with CAFs. Statistical significance of *n* = 3 replicas was calculated using one‐way ANOVA, combined with Dunnett's test with respective untreated control as reference in each model system (*p < 0.05, **p < 0.01, ***p < 0.001, ****p < 0.0001). (C) Representative phase‐contrast images of LNCaP 2D cultures with or without GFP‐tagged CAFs. Scale bars 100 μm. (D) In parallel, LNCaP cells were cultured in 3D organotypic conditions, using mixed Matrigel/collagen type I gels. Representative images of 3D mono‐ and co‐cultures of LNCaP with CAFs expressing GFP are shown. Scalebars 200 μm. (E) Spinning disk confocal microscopy images with 40× objective labeled by immunofluorescence with an antibody against α‐smooth muscle actin (α‐actin, green), which serves as a marker of cancer‐associated fibroblasts (indicated with arrows), epithelial cell marker (cytokeratin 8 + 18, red), and nuclear counterstain (Draq5, blue). Scale bars 100 μm.

For 2D co‐cultures, different ratios of cancer cells and CAFs were tested to identify optimal conditions for reproducible and stable tumor/stroma co‐cultures. CAFs and PCa cell lines (LNCaP, VCaP) were co‐cultured at defined ratios and grown as a 2D monolayer. The metabolic assay indicating cellular viability of the cancer cells and CAFs together, confirmed the pronounced and consistent protective effect of CAFs against growth inhibition by FGFRi (Figure 2A), in comparison to cancer cell mono‐cultures (Figure 2A,C). The protective effect of CAFs against FGFRi was more pronounced in LNCaP with CAF co‐cultures, compared to VCaP with CAF co‐cultures (Figure 2A and Figure S2).

In parallel, we tested tumor/stroma co‐cultures in organotypic 3D culture conditions, embedded in a Matrigel/collagen type I matrix using the “sandwich model”.^34^, ^46^ Unfortunately, VCaP cells failed to spontaneously form organotypic growths in Matrigel/collagen gels and could not be tested in 3D culture. Treatment effects on cell viability of the 3D co‐cultured cancer cells were significantly less pronounced in the presence of CAFs (Figure 2B). These effects are also visualized in phase contrast microscopy images, indicating reduced growth‐inhibitory effects in 3D co‐cultures in the presence of CAFs (green) compared to 3D mono‐cultures (Figure 2D). In the absence of CAFs, LNCaP organotypic structures treated with FIIN1 or FIIN2 were significantly smaller, more irregular, and showed a more uneven, rough surface (Figure 2D,E). Figure 2E is a higher magnification image showing the green CAF distribution and morphology. The CAF marker, α‐smooth muscle actin, and epithelial cancer cell marker cytokeratin 8 + 18 are detectable after drug treatment in the 3D co‐culture setting.

### 3.4 The FRS2αi targeting the downstream p‐FRS2α is more specific and effective against FGFR‐dependent PCa cells than FIIN1 and FIIN2

**FIGURE 3:**
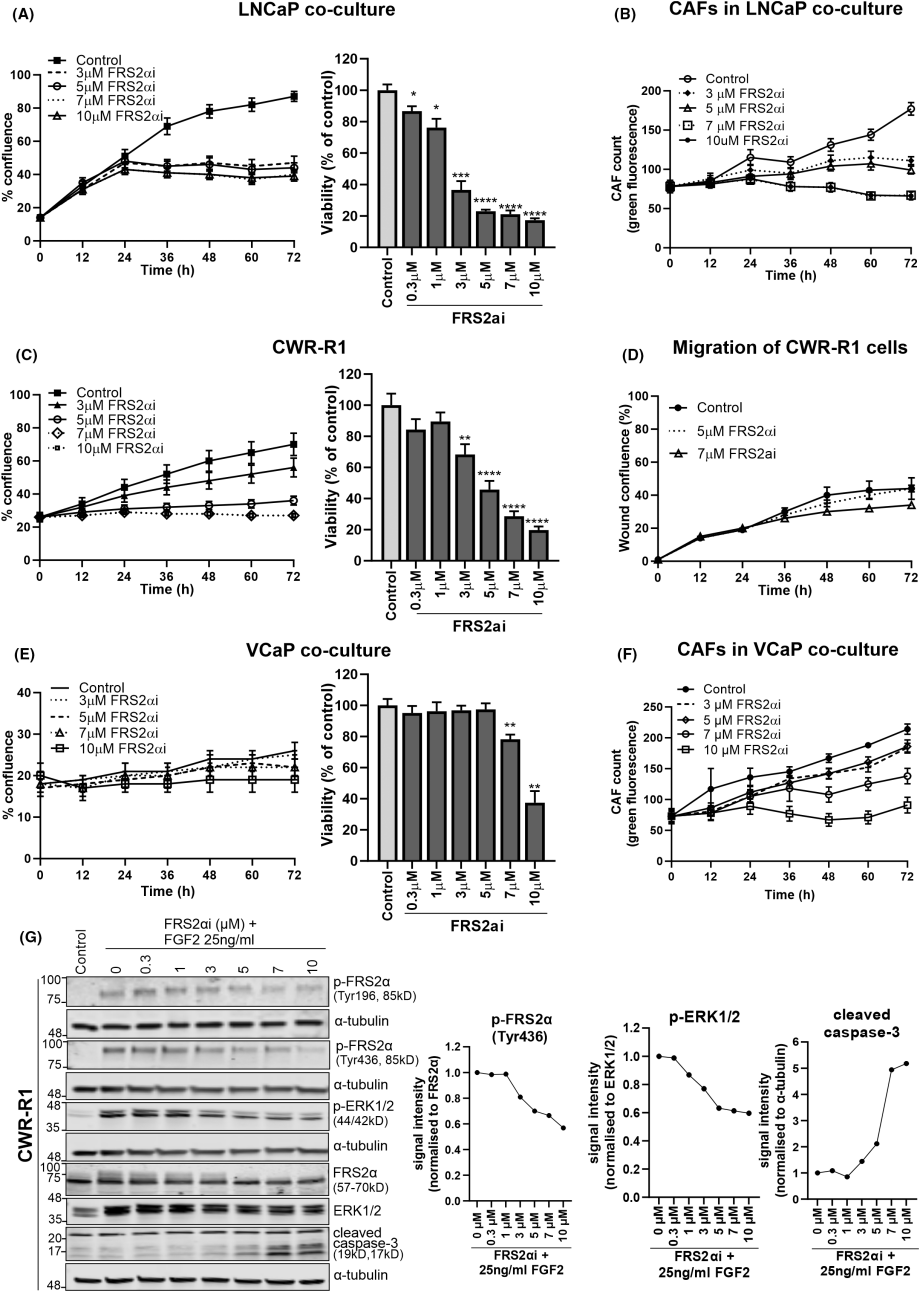
The FRS2αi blocks cancer cells and CAFs growth in a dose‐dependent manner in the PCa co‐cultures of LNCaP, VCaP, and CWR‐R1. (A, C, E) Time course effects of FRS2αi treatment on proliferation (line graphs) and cellular viability (bar graphs), expressed as percentage of solvent (0.1% DMSO) control in LNCaP, CWR‐R1, and VCaP co‐cultures with CAFs. (B, D, F) Time course analysis of FRS2αi treatment effects on the growth of CAFs expressed as the count of green fluorescent cells in (B, F) and migration of the cells into the wound expressed as the percentage of wound confluence in (D). Statistical significance of *n* = 3 replicates in A, B, and C were calculated using one‐way ANOVA using Dunnett's test with controls (0.1% DMSO) as reference (*p < 0.05, **p < 0.01, ***p < 0.001, ****p < 0.0001). (G) Immunoblot analysis of FGFR downstream signaling, with protein band signal intensities, indicated in the line graphs in response to 0 μM–10 μM FRS2αi treatment. Antibodies used are indicated to the right of the immunoblot panels.

Since even the second‐generation FGFRis, FIIN1, and FIIN2 showed mostly unspecific and relatively mild effects on PCa co‐cultures, we explored an experimental inhibitor of the FRS2a signaling molecule (“compound 7”, named FRS2αi^44^). LNCaP co‐culture and CWR‐R1 cells were both sensitive to FRS2αi in the range between 0.3 μM and 10 μM, effectively inhibiting proliferation (indicated by the lower percentage of confluence in the line graphs of Figure 3A–C) leading to a decrease in metabolic activity indicated as cell viability (bar graphs of Figure 3A,C). Moreover, the migration of the CWR‐R1 cells also decreased upon treatment with 7 μM FRS2αi (Figure 3D). In contrast, VCaP co‐culture with CAFs only responded to high concentrations (>7 μM) of FRS2αi, lacking the characteristic dose‐dependent sensitivity for the lower range of concentrations (0.3 μM–5 μM, Figure 3E). The VCaP doubling time is almost twice that of LNCaP and CWR‐R1, which may account for the relatively small increase in the confluence of VCaP control (Figure 3E). A detailed image analysis using the IncuCyte S3 imager and software showed that the FRS2αi also decreased the proliferation of the CAFs in the co‐culture settings in a dose‐dependent manner, but more than 25% of the CAFs persisted after 72 h treatment even at the highest drug concentrations (10 μM) of FRS2αi (Figure 3B,F). These results identify FRS2αi as a potential drug candidate that more specifically targets activated FGFR signaling in PCa cells than second generation FGFRi, without showing excessive toxicity on stromal cells such as fibroblasts.

The molecular effects of FRS2αi on key signaling molecules in PCa cells were further investigated (Figure 3G). FRS2αi treatment at concentrations greater than 5 μM reduced the phosphorylation at both the Tyr196 and the Tyr436 sites of FRS2α and the phosphorylation of ERK1/2 (line graphs of Figure 3G). Exposure of cells to this compound also led to increased levels of cleaved caspase‐3 subunits p19 and p17, indicating induction of apoptosis, and reduction in the overall confluence of the cells.

### 3.5 Treatment effects of FIIN1, FIIN2, and FRS2αi on proliferation and viability of CAF mono‐cultures

**FIGURE 4:**
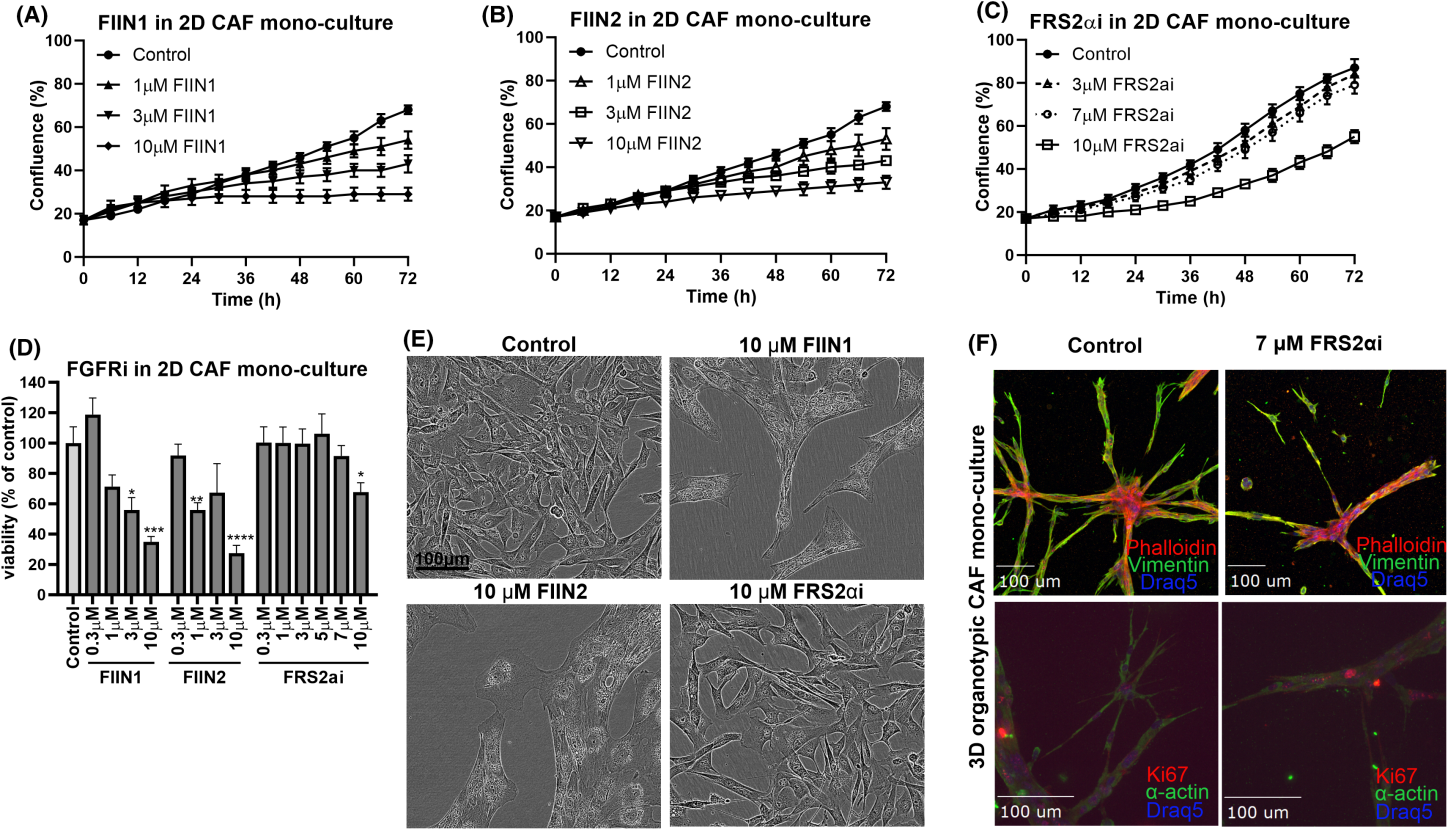
The FGFRis affect proliferation of CAFs in a dose‐dependent manner (A–C). Time course shows the effects of FIIN1 (A), FIIN2 (B), and FRS2αi (C) treatment on proliferation (indicated as percentage of confluence) and (D) cell viability after 72 h of treatment, expressed as the percentage of solvent (0.1% DMSO) control. Statistical significance of *n* = 3 replicas was calculated using one‐way ANOVA and Dunnett's test with controls (0.1% DMSO) as a reference (*p < 0.05, **p < 0.01, ***p < 0.001, ****p < 0.0001). (E) Representative phase‐contrast images of CAF 2D mono‐cultures after 72 h treatment with FGFRis. Scale bar 200 μm. (F) Immunofluorescent staining of CAFs in 3D organotypic culture against mesenchymal markers (vimentin), F‐Actin (Phalloidin), a cell proliferation marker (Ki67), and nuclear DNA counterstain (Draq5) after 6 days of treatment with FRS2αi. The images were captured with a 40× objective using the spinning disk confocal microscope. Scalebar = 100 μm.

The FGFRi treatment on CAF mono‐cultures led to a concentration‐dependent reduction in the proliferation of the CAFs (Figure 4A–C). The viability of CAFs was significantly affected at a concentration of 10 μM for all three FGFRi and decreased the confluence of the CAFs by 50% after 72 h of treatment (Figure 4C). In contrast, 3 μM and 7 μM of FRS2αi had much milder effects on proliferation and lower cytotoxicity as compared to FIIN1 and FIIN2 (Figure 4D). The representative phase‐contrast images of the density and the morphology of CAFs after 72 h of treatment are shown in Figure 4E. The lower cytotoxicity of FRS2αi and increased specificity to block active FGFR signaling (as mentioned in section 3.4) is further indicated by treatment of the CAFs in 3D organotypic cultures. Here, CAFs persist even after 6 days of exposure to 7 μM FRS2αi, retain stable expression of the mesenchymal marker vimentin, and still proliferate as indicated by expression of Ki67 marker (Figure 4F).

### 3.6 FRS2αi treatment of 3D organotypic co‐cultures with CAFs affects the contact between CAFs and cancer cell clusters

**FIGURE 5:**
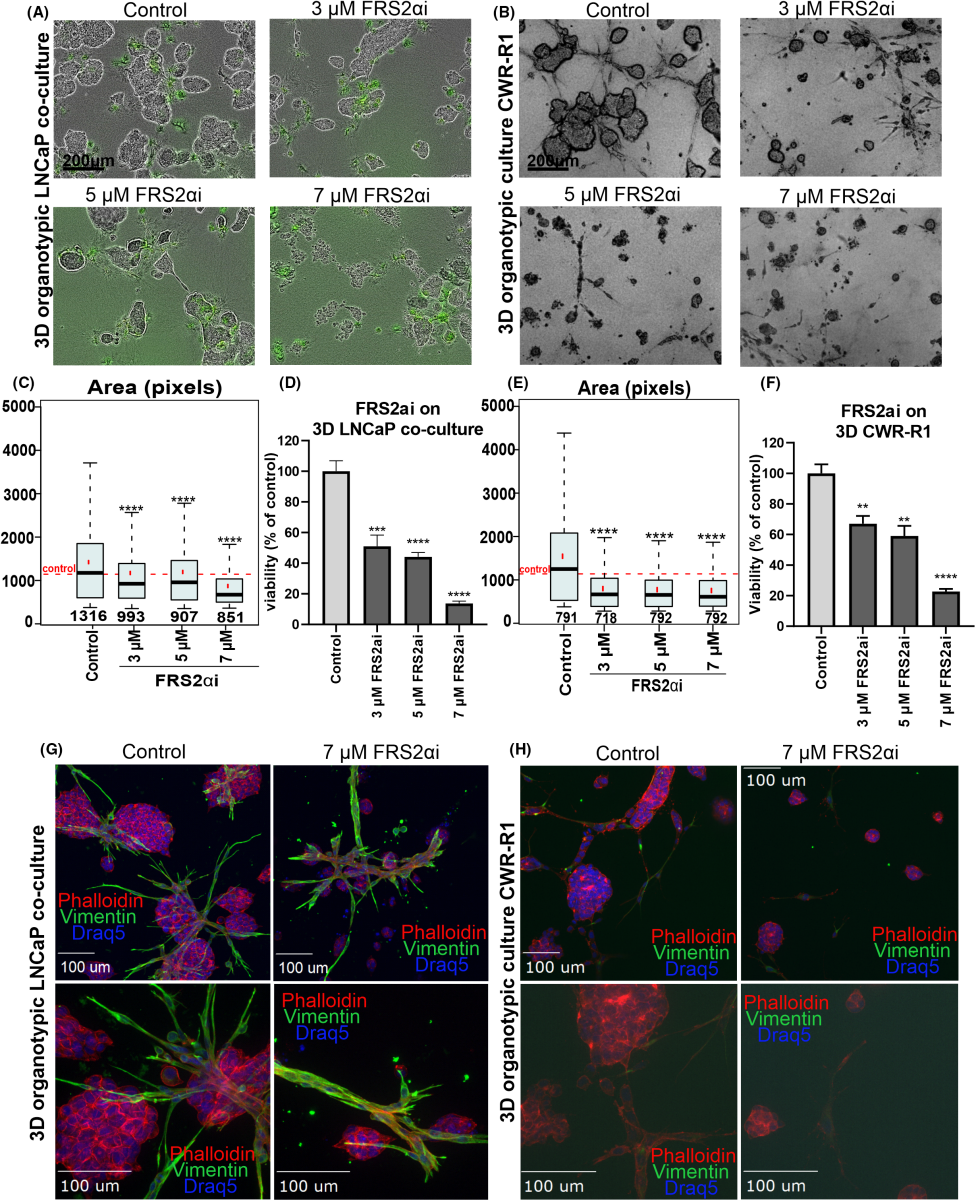
FRS2αi represses PCa growth in 3D‐organotypic co‐cultures with CAFs. (A) Representative phase contrast and green fluorescence (GFP) images of organotypic co‐cultures of LNCAP + CAF. (B) Brightfield microscope images of CWR‐R1 at the endpoint of treatment with FRS2αi. Scale bar of 200 μm. (C–F) Quantitative analysis of size and viability 3D LNCaP co‐cultures (C, D) and CWR‐R1 (E,F). The box and whisker plots represent the median size of the treated organotypic co‐cultures (black horizontal line), the median size of the controls (dotted red horizontal line) and the total number of objects in the analyses. Statistical significance of *n* = 3 replica calculated using Bonferroni‐corrected *t*‐test (*p < 0.05, **p < 0.01, ***p < 0.001, ****p < 0.0001). The viability analysis of LNCaP co‐culture organotypic growth indicated in (D) and CWR‐R1 in (F), exposed to FRS2αi. Statistical analysis with one‐way ANOVA using Dunnett's test of *n* = 3 replicas (*p < 0.05, **p < 0.01, ***p < 0.001, ****p < 0.0001). (G, H) Immunofluorescence staining of 3D co‐cultures after 6 days of treatment with 7 μM FRS2αi in LNCaP with CAFs (G) and CWR‐R1 (H) with CAF specific expression protein, a mesenchymal marker (vimentin), F‐actin (Phalloidin), and nuclear DNA counterstain (Draq5). The images were captured with 20× and 40× objectives using the spinning disk confocal microscope. Scalebar = 100 μm.

The effects of compound FRS2αi were tested in 3D organotypic co‐cultures of CWR‐R1 and LNCaP cells with CAFs. These CAFs typically surround and interact closely with the cancer cells (as seen in Figure 2E and Figure S3). We assume that the close contact of tumor cells to CAFs likely activates several autocrine, juxtracrine, and paracrine support functions including FGFR signaling. The 3D organotypic co‐cultures were precultured for 4 days and treated with FRS2αi (3 μM−7 μM) for 6 days (Figure 5). The live‐cell images show that FRS2αi treatment significantly decreased the size of the organotypic cultures, while the proliferation and number of CAFs were affected but not completely lost at the endpoint of treatment (Figure 5A,B). The quantified effects of the treatment are presented as box and whisker plots reflecting a significant decrease in organotypic structure size [measured as the *area* in pixels, (Figure 5C,E)], and the bar graphs show a significant reduction of their viability (Figure 5D,F). Additionally, immunofluorescent images with higher magnification show a decrease in direct contact between several cancer cell clusters and CAFs. Cancer cell clusters that have retained contact with CAFs after treatment with FRS2αi also show growth inhibition based on their smaller size compared to the untreated controls (Figure 5G,H).

### 3.7 Combinatorial treatment of FRS2αi with ARi in 2D and 3D co‐cultures with CAFs does not show significant added or synergistic effects over single treatment with FRS2αi

Optimized concentrations of R1881 showed effective activation of AR target genes in VCaP and LNCaP cell lines,^48^ including prostate‐specific antigen (PSA). In contrast, the CWR‐R1 cell line does not express and secrete PSA but is characterized by hyperactive AR signaling that results in androgen‐independent growth which is not responsive to androgen deprivation.^31^, ^32^ This is shown by the persistent protein expression of AR itself, and stable AR‐driven expression of target genes like calcium/calmodulin‐dependent protein kinase kinase 2 (CAMKK2) in CWR‐R1 cells even with ARi treatment (Figure S4A). In LNCaP and VCaP cells, treatment with ARi, enzalutamide or darolutamide reduced the transcriptional androgen response, such as PSA expression, already at 1 μM concentration (Figure S4A). Therefore, 1 μM of these ARis were used as the minimally effective concentration that results in significant inhibition of AR‐dependent target protein expression. This was combined with 3 μM–7 μM of FRS2αi and tested in both 2D and 3D co‐cultures of CWR‐R1, LNCaP, and VCaP cells with CAFs. Exposure to FRS2αi alone, effectively blocked cell proliferation (as shown in section 3.4 and Figure 3). However, no significant change in proliferation over FRS2αi alone was observed upon combinatorial treatments with ARi in 2D co‐cultures for both LNCaP (Figures S5A, S6A) and CWR‐R1 cells (Figures S5C, S6C). As expected, only weak effects of FRS2αi with ARi on cell proliferation were observed for VCaP co‐culture (Figures S5B, S6B). Correspondingly, the cellular viability upon combinatorial treatment with FRS2αi and ARi, had no additive or synergistic effects (Figures S5D, S6D) in 2D co‐cultures. The single and combinatorial effects on CAFs quantitated by distinct, separate image analysis and segmentation of GFP‐labeled cells in the co‐culture were not significantly different (Figure S7). Similar observation of lack of benefit upon this combination of 3 μM–7 μM FGFRi and 1 μM ARi was observed in 3D organotypic co‐cultures as well (Figures S8A, S9A for 3D LNCaP co‐cultures and Figures S8B, S9B for 3D CWR‐R1 cultures). The quantifications of the treated 3D co‐cultures (Figures S8C–F, S9C–F) reiterate the lack of synergistic effect of the combinatorial treatment in growth response (measures as *area* in pixels), cytotoxicity (measured as % *roundness* of the organotypic culture), invasiveness [measured as maximum length of appendages (*MaxApp* in pixels)], and viability of the cells. These morphometric treatment effects were quantified by segmentation of the 3D structures using the AMIDA image analysis tool (Figure S10).

### 3.8 Anti‐proliferative effect of FRS2αi on CAFs and cancer cells leading to inhibition of growth in 3D organotypic co‐cultures

**FIGURE 6:**
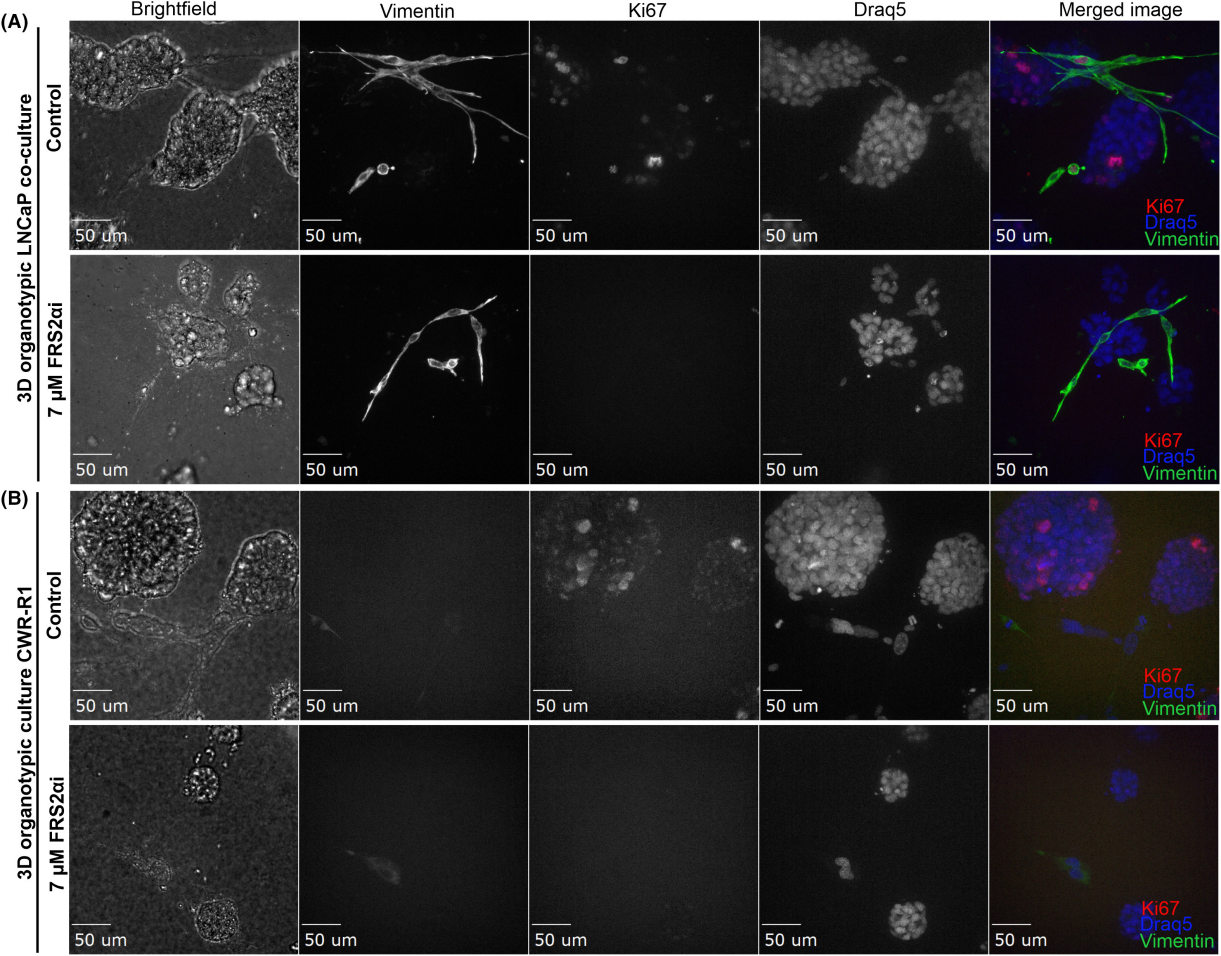
Immunofluorescence staining of (A) LNCaP with CAFs and (B) CWR‐R1 organotypic 3D co‐cultures after 6 days of treatment with 7 μM FRS2αi. The 3D co‐cultures were stained for vimentin (CAF marker, green channel), Ki67 (proliferation marker, red channel), and Draq5 (nuclei, blue channel). The merged Z‐stack images from confocal microscopy are displayed in greyscale and shown in the merged images in color. Scale bar = 50 μm.

In the 3D organotypic co‐cultures, CAFs and cancer cells are in physical contact with each other, which supports the autocrine and paracrine activation of FGFR signaling. The CAFs actively interact and grow along with the epithelial cancer cells as organotypic cultures (Figure 6A,B control group). The FRS2αi treatment of 3D co‐cultures of LNCaP and CWR‐R1 significantly reduced the growth of the tumor cells, while the decreased proliferation of CAFs results in a gradual destabilization of the tumor cell‐stroma interaction, which causes a loss of cellular integrity within the organotypic structures. This is shown by the decreased expression of cell proliferation marker Ki67 (Figure 6A for LNCaP co‐cultures and Figure 6B for CWR‐R1). The number of nuclei within the 3D structures was also decreased as shown in the Draq5 nuclear staining (Figure 6), again indicating the anti‐proliferative effect of FRS2αi.

## 4 DISCUSSION

The first‐generation receptor tyrosine kinase inhibitors (RTKis) are less potent against FGFRs and are unspecific as they also target PDGFRs and VEGFRs. The second‐generation, pan‐FGFR, non‐covalent FGFRi such as fexagratinib/AZD4547, infigratinib/BGJ398, and erdafitinib/JNJ‐42756493 have largely failed to provide encouraging results in clinical trials in PCa.^49^, ^50^, ^51^ This is likely due to the cancer cells acquiring resistance through FGFR gatekeeper mutations that block the initial inhibitory activity of these compounds. However, this is the first study that explores the action of the second‐generation, covalent FGFRi (FIIN1 and FIIN2) that overcome the resistance to gatekeeper mutations,^38^, ^39^, ^40^ and the activity of a novel downstream FRS2αi.^44^

The FGFR 1–4 are expressed in LNCaP, VCaP, and CWR‐R1 cells but respond to FGF2 induction only in LNCaP and CWR‐R1 by a specific downstream mediator of FGFR signaling, fibroblast growth factor receptor substrate 2α (FRS2α). In response to FGF2 exposure, FRS2α is rapidly phosphorylated at Tyr196 and Tyr436 in CWR‐R1 cells, but only at Tyr196 in LNCaP cells. In contrast, FRS2α is not measurably phosphorylated upon FGF2 exposure in VCaP cells. These observations essentially show that VCaP cells are likely not sensitive to FGFR signaling, whereas LNCaP cells are still responsive, and CWR‐R1 cells are the most responsive and possibly dependent on FGFR signaling. The FGFRi, FIIN1, and FIIN2 effectively decrease FGFR downstream signaling in LNCaP and VCaP even at lower concentrations around 0.3 μM with complete inhibition of p‐ERK1/2 unlike in CWR‐R1. We interpret this as unspecific drug activity that may also target other tyrosine‐kinase receptors such as EGFR.^40^ It has been shown previously and in our data that the three cell lines used in this study are AR‐positive with CWR‐R1 being androgen‐independent, LNCaP is androgen‐sensitive and VCaP is androgen‐dependent.^52^ The EGFR pathway is regulated by androgens in AR‐dependent cells, explaining the unspecific drug activity of FIIN1/2, particularly in VCaP.^53^, ^54^, ^55^

CAFs are an essential part of the TME, and potently promote and support tumor growth and progression in PCa.^21^, ^56^ To mimic various signaling aspects of the TME, mono‐culture of cancer cells versus co‐cultures with CAFs were used as 3D organotypic models. In earlier studies, CAFs were already shown to increase chemoresistance in 2D co‐cultures.^22^, ^57^, ^58^ Likewise, the co‐cultures of CAFs with LNCaP cells significantly lowered the sensitivity to FGFRis. In contrast, the protective effect of CAFs on VCaP cells in 2D co‐culture was only minimal, which reiterates that VCaP cells are likely not dependent on FGF‐FGFR signaling for growth and proliferation.

The FRS2αi was most effective in FGFR‐dependent co‐cultures of CWR‐R1 and LNCaP cells with CAFs, which are responsive to FGF2 and display activated p‐FRS2α. At the molecular level, the FRS2αi modestly inhibited Tyr196 and Tyr436 phosphorylation sites in FRS2α and ERK1/2 phosphorylation at a concentration of 3 μM and above. Nevertheless, this inhibition was sufficient to significantly reduce proliferation and cell viability through apoptosis as shown by the increase of cleaved caspase‐3. The only moderately reduced phosphorylation of FRS2α at Tyr196 and Tyr436 even at higher concentrations of FRS2αi—despite the detected biological activity—could be explained by FRS2αi inhibition of any other of the four additional tyrosine phosphorylation sites of FRS2α,^59^ for which we were unable to obtain antibodies.

Previous studies have also shown that CWR‐R1 is a model for androgen‐independent CRPC that has a high expression of truncated AR variants.^31^, ^32^ CWR‐R1 cells may actively bypass AR signaling by using the FGFR pathway for survival, growth, and proliferation. LNCaP cells are sensitive to (but not dependent on) androgens and to FGFR signaling. In contrast, VCaP cells appeared to strongly depend on continuous AR signaling for cell proliferation and survival.^29^ In combinatorial treatment with FRS2αi, ARi (enzalutamide, darolutamide) was neither additive nor synergistic in any of the cell lines tested. Another recent study with FGFR‐dependent CRPC also showed little benefit of combination treatment over single treatment with FGFRi, erdafitinib, and enzalutamide.^60^

Since the effects of the FRS2αi were prominent in two of the three PCa cell lines tested, we tested its effects on proliferation and viability also in 3D co‐cultures with CAFs. In 3D co‐culture, cellular interactions and signaling are strongly facilitated by the presence of CAFs. Importantly, our organotypic‐associated CAFs express α‐smooth muscle actin (α‐SMA) and vimentin which suggests that these are indeed tumor‐promoting CAFs, and not normal fibroblasts (NCAF), or the type of fibroblasts associated with benign prostate‐hyperplasia (BPH).^21^, ^61^, ^62^ Previous studies showed that CAFs in physical, close contact with the tumor cells (as also observed in this study) are often pro‐inflammatory. These pro‐inflammatory CAFs were shown to stimulate tumor growth, while other CAF subtypes, which are also typically further away from the tumor cells, are likely to be anti‐inflammatory.^63^, ^64^, ^65^, ^66^ The exposure to the FRS2αi effectively reduced the proliferation of tumor‐promoting CAFs that were in physical contact with the tumor cells, resulting in the disruption of viable tumor‐stroma interactions, and decreasing the proliferation of the tumor organotypic structures as indicated by the significant reduction of Ki67. Unlike the second generation, irreversible inhibitors, FIIN1 and FIIN2; the FRS2αi treatment was not overly cytotoxic to CAFs below a concentration of 10 μM. It likely indicates that this compound specifically represses cancer‐promoting functions that depend on active FGF‐FGFR interactions between CAF and cancer cells. Altogether, FRS2αi represents a specific and effective agent for specifically blocking the growth of FGFR‐dependent PCa cells.

## 5 CONCLUSION

The FGFR pathway is a clinically relevant target that contributes to cancer growth in treatment‐refractory metastatic CRPC, particularly in double negative (AR negative and neuroendocrine negative) PCa.^6^, ^60^ In combination with cancer cells, CAFs provide a more physiologically representative in vitro model in drug screening for FGFRis. The next‐generation FGFRis tested in this study, particularly FRS2αi proved to be anti‐proliferative and induce apoptosis in co‐cultures with activated FGFR signaling but did not have much effect in cells that are less dependent on FGFR signaling for growth. Overall, the molecular characterization of the tumor signaling network and vulnerabilities, including FGFR pathway activity, are critical to determining an effective strategy for treatment decisions in metastatic CRPC.

## AUTHOR CONTRIBUTIONS

**Syeda Afshan:** Conceptualization (lead); data curation (lead); formal analysis (lead); funding acquisition (equal); investigation (lead); methodology (lead); project administration (equal); resources (supporting); software (equal); supervision (supporting); validation (lead); visualization (lead); writing – original draft (lead); writing – review and editing (lead). **Yu Gang Kim:** Investigation (supporting); methodology (supporting); validation (supporting). **Jesse Mattsson:** Formal analysis (supporting); software (equal). **Malin Åkerfelt:** Methodology (supporting); supervision (supporting); writing – review and editing (supporting). **Pirkko Härkönen:** Funding acquisition (equal); project administration (equal); resources (equal); supervision (equal); writing – review and editing (supporting). **Martin Baumgartner:** Funding acquisition (supporting); resources (supporting); writing – review and editing (supporting). **Matthias Nees:** Conceptualization (equal); funding acquisition (equal); project administration (equal); resources (lead); supervision (lead); visualization (supporting); writing – original draft (supporting); writing – review and editing (supporting).

## FUNDING INFORMATION

The research was supported by funds from the Turku University Foundation/Turun Yliopistosäätiö (080956, S.A.), the Finnish‐Norwegian Medical Foundation/Suomalais‐Norjalainen Lääketieteen Säätiö (2022043, S.A.), the Southwest Finland Cancer Organization/Lounaissuomalaiset Syöpäjärjestöt (S.A.), Ida Montinin Säätiö, Finland (20230339, S.A.), the Drug Research Doctoral Program of the University of Turku, Finland (S.A.), the Paulo Foundation/Paulon Säätiö, Finland (S.A.), the Swiss National Science Foundation (SNF_310030_188793, M.B.), the Swiss Cancer Research Foundation (KFS‐4853‐08‐2019, M.B.), Academy of Finland (267326, 309372; M.N., P.H.), the European Union's TransPot Horizon 2020 research and innovation programme under the Marie Skłodowska‐Curie grant agreement (721746, M.N., P.H., S.A.) and open access funding provided by University of Turku (UTU).

## CONFLICT OF INTEREST STATEMENT

The authors declare that they have no conflicts of interest.

## ETHICS STATEMENT

This study has used commercially available cell lines from ATCC as listed in section 2.1 of this article. ATCC obtains informed consent from the originators of the cell lines. The use of these cell lines for research purposes does not require additional ethical approvals from the ethics committee of Southwest Finland.

## Supporting information


Figures S1–S10.

Table S1.

## Data Availability

The authors confirm that the data supporting the findings of this study are available within the article and its supplementary materials.

## References

[1] Mills IG . Maintaining and reprogramming genomic androgen receptor activity in prostate cancer. Nat Rev Cancer. 2014;14(3):187‐198. doi:10.1038/nrc3678 24561445

[2] Poutanen M , Hagberg Thulin M , Härkönen P . Targeting sex steroid biosynthesis for breast and prostate cancer therapy. Nat Rev Cancer. 2023;23:686‐709. doi:10.1038/s41568-023-00609-y 37684402

[3] Mori K , Mostafaei H , Pradere B , et al. Apalutamide, enzalutamide, and darolutamide for non‐metastatic castration‐resistant prostate cancer: a systematic review and network meta‐analysis. Int J Clin Oncol. 2020;25:1892‐1900. doi:10.1007/s10147-020-01777-9 32924096 PMC7572325

[4] Watson PA , Arora VK , Sawyers CL . Emerging mechanisms of resistance to androgen receptor inhibitors in prostate cancer. Nat Rev Cancer. 2015;15(12):701‐711. doi:10.1038/nrc4016 26563462 PMC4771416

[5] Kirby M , Hirst C , Crawford ED . Characterising the castration‐resistant prostate cancer population: A systematic review. Int J Clin Pract. 2011;65(11):1180‐1192. doi:10.1111/j.1742-1241.2011.02799.x 21995694

[6] Bluemn EG , Coleman IM , Lucas JM , et al. Androgen receptor pathway‐independent prostate cancer is sustained through FGF signaling. Cancer Cell. 2017;32(4):474‐489.e6. doi:10.1016/j.ccell.2017.09.003 29017058 PMC5750052

[7] Berger A , Brady NJ , Bareja R , et al. N‐Myc‐mediated epigenetic reprogramming drives lineage plasticity in advanced prostate cancer. J Clin Invest. 2019;129(9):3924‐3940. doi:10.1172/JCI127961 31260412 PMC6715370

[8] Kähkönen TE , Ivaska KK , Jiang M , et al. Fibroblast growth factor receptors (Fgfrs): structures and small molecule inhibitors. Sci Rep. 2019;12(1):112‐121. doi:10.1074/jbc.M601248200 PMC6627960 31216761

[9] Yu L , Toriseva M , Afshan S , et al. Increased expression and altered cellular localization of fibroblast growth factor receptor‐like 1 (FGFRL1) are associated with prostate cancer progression. Cancer. 2022;14(2):278. doi:10.3390/cancers14020278 PMC8796033 35053442

[10] Kwabi‐Addo B , Ozen M , Ittmann M . The role of fibroblast growth factors and their receptors in prostate cancer. Endocr Relat Cancer. 2004;11(4):709‐724. doi:10.1677/erc.1.00535 15613447

[11] Rosini P , Bonaccorsi L , Baldi E , et al. Androgen receptor expression induces FGF2, FGF‐binding protein production, and FGF2 release in prostate carcinoma cells: role of FGF2 in growth, survival, and androgen receptor down‐modulation. Prostate. 2002;53(4):310‐321. doi:10.1002/pros.10164 12430142

[12] Zhou W , Su Y , Zhang Y , Han B , Liu H , Wang X . Endothelial cells promote docetaxel resistance of prostate cancer cells by inducing ERG expression and activating Akt/mTOR signaling pathway. Front Oncol. 2020;10:584505. doi:10.3389/fonc.2020.584505 33425737 PMC7793734

[13] Wesche J , Haglund K , Haugsten EM . Fibroblast growth factors and their receptors in cancer. Biochem J. 2011;437(2):199‐213. doi:10.1042/BJ20101603 21711248

[14] Parker BC , Engels M , Annala M , Zhang W . Emergence of FGFR family gene fusions as therapeutic targets in a wide spectrum of solid tumours. J Pathol. 2014;232(1):4‐15. doi:10.1002/path.4297 24588013

[15] Giacomini A , Grillo E , Rezzola S , et al. The FGF/FGFR system in the physiopathology of the prostate gland. Physiol Rev. 2020;101:569‐610. doi:10.1152/physrev.00005.2020 32730114

[16] Elo T , Sipilä P , Valve E , et al. Fibroblast growth factor 8b causes progressive stromal and epithelial changes in the epididymis and degeneration of the seminiferous epithelium in the testis of transgenic mice. Biol Reprod. 2012;86(5):157. doi:10.1095/biolreprod.111.097352 22423049

[17] Corn PG , Wang F , McKeehan WL , Navone N . Targeting fibroblast growth factor pathways in prostate cancer. Clin Cancer Res. 2013;19(21):5856‐5866. doi:10.1158/1078-0432.CCR-13-1550 24052019 PMC3926427

[18] Chen S , Zhu G , Yang Y , et al. Single‐cell analysis reveals transcriptomic remodellings in distinct cell types that contribute to human prostate cancer progression. Nat Cell Biol. 2021;23(1):87‐98. doi:10.1038/s41556-020-00613-6 33420488

[19] Li H , Zhang J , Chen SW , et al. Cancer‐associated fibroblasts provide a suitable microenvironment for tumor development and progression in oral tongue squamous cancer. J Transl Med. 2015;13:198. doi:10.1186/s12967-015-0551-8 26094024 PMC4475624

[20] Madar S , Goldstein I , Rotter V . “Cancer associated fibroblasts”—more than meets the eye. Trends Mol Med. 2013;19(8):447‐453. doi:10.1016/j.molmed.2013.05.004 23769623

[21] Neuwirt H , Bouchal J , Kharaishvili G , et al. Cancer‐associated fibroblasts promote prostate tumor growth and progression through upregulation of cholesterol and steroid biosynthesis. Cell Commun Signal. 2020;18(1):11. doi:10.1186/s12964-019-0505-5 31980029 PMC6979368

[22] Kuzet SE , Gaggioli C . Fibroblast activation in cancer: When seed fertilizes soil. Cell Tissue Res. 2016;365(3):607‐619. doi:10.1007/s00441-016-2467-x 27474009

[23] Richards Z , McCray T , Marsili J , et al. Prostate stroma increases the viability and maintains the branching phenotype of human prostate organoids. iScience. 2019;12:304‐317. doi:10.1016/j.isci.2019.01.028 30735898 PMC6365938

[24] Haffner MC , Zwart W , Roudier MP , et al. Genomic and phenotypic heterogeneity in prostate cancer. Nat Rev Urol. 2021;18(2):79‐92. doi:10.1038/s41585-020-00400-w 33328650 PMC7969494

[25] Servant R , Garioni M , Vlajnic T , et al. Prostate cancer patient‐derived organoids: detailed outcome from a prospective cohort of 81 clinical specimens. J Pathol. 2021;254:543‐555. doi:10.1002/path.5698 33934365 PMC8361965

[26] Drost J , Karthaus WR , Gao D , et al. Organoid culture systems for prostate epithelial and cancer tissue. Nat Protoc. 2016;11:347‐358. doi:10.1038/nprot.2016.006 26797458 PMC4793718

[27] Ayuso JM , Vitek R , Swick AD , et al. Effects of culture method on response to EGFR therapy in head and neck squamous cell carcinoma cells. Sci Rep. 2019;9(1):12480. doi:10.1038/s41598-019-48764-3 31462653 PMC6713778

[28] Åkerfelt M , Bayramoglu N , Robinson S , et al. Automated tracking of tumor‐stroma morphology in microtissues identifies functional targets within the tumor microenvironment for therapeutic intervention. Oncotarget. 2015;6:30035‐30056. doi:10.18632/oncotarget.5046 26375443 PMC4745780

[29] Moilanen AM , Riikonen R , Oksala R , et al. Discovery of ODM‐201, a new‐generation androgen receptor inhibitor targeting resistance mechanisms to androgen signaling‐directed prostate cancer therapies. Sci Rep. 2015;5:12007. doi:10.1038/srep12007 26137992 PMC4490394

[30] Syvälä H , Pennanen P , Bläuer M , Tammela TLJ , Murtola TJ . Additive inhibitory effects of simvastatin and enzalutamide on androgen‐sensitive LNCaP and VCaP prostate cancer cells. Biochem Biophys Res Commun. 2016;481(1–2):46‐50. doi:10.1016/j.bbrc.2016.11.021 27833018

[31] Nyquist MD , Dehm SM . Interplay between genomic alterations and androgen receptor signaling during prostate cancer development and progression. Horm Cancer. 2013;4:61‐69. doi:10.1007/s12672-013-0131-4 23307762 PMC3957092

[32] Dehm SM , Schmidt LJ , Heemers HV , Vessella RL , Tindall DJ . Splicing of a novel androgen receptor exon generates a constitutively active androgen receptor that mediates prostate cancer therapy resistance. Cancer Res. 2008;68(13):5469‐5477. doi:10.1158/0008-5472.CAN-08-0594 18593950 PMC2663383

[33] Gnanapragasam VJ , Robson CN , Neal DE , Leung HY . Regulation of FGF8 expression by the androgen receptor in human prostate cancer. Oncogene. 2002;21(33):5069‐5080. doi:10.1038/sj.onc.1205663 12140757

[34] Härmä V , Schukov HP , Happonen A , et al. Quantification of dynamic morphological drug responses in 3D organotypic cell cultures by automated image analysis. PLoS One. 2014;9(5):e96426. doi:10.1371/journal.pone.0096426 24810913 PMC4014501

[35] Björk JK , Åkerfelt M , Joutsen J , et al. Heat‐shock factor 2 is a suppressor of prostate cancer invasion. Oncogene. 2016;35:1770‐1784. doi:10.1038/onc.2015.241 26119944 PMC4830906

[36] Ahonen I , Härmä V , Schukov H‐P , Nees M , Nevalainen J . Morphological clustering of cell cultures based on size, shape and texture features. Stat Biopharm Res. 2016;8(2):217‐228. doi:10.1080/19466315.2016.1146162

[37] Madar S , Brosh R , Buganim Y , et al. Modulated expression of WFDC1 during carcinogenesis and cellular senescence. Carcinogenesis. 2009;30(1):20‐27. doi:10.1093/carcin/bgn232 18842679 PMC2639035

[38] Huang Z , Tan L , Wang H , et al. DFG‐out mode of inhibition by an irreversible type‐1 inhibitor capable of overcoming gate‐keeper mutations in FGF receptors. ACS Chem Biol. 2015;10(1):299‐309. doi:10.1021/cb500674s 25317566 PMC4301177

[39] Zhou W , Hur W , McDermott U , et al. A structure‐guided approach to creating covalent FGFR inhibitors. Chem Biol. 2010;17(3):285‐295. doi:10.1016/j.chembiol.2010.02.007 20338520 PMC2920453

[40] Tan L , Wang J , Tanizaki J , et al. Development of covalent inhibitors that can overcome resistance to first‐generation FGFR kinase inhibitors. Proc Natl Acad Sci USA. 2014;111(45):E4869‐E4877. doi:10.1073/pnas.1403438111 25349422 PMC4234547

[41] Fumarola C , Bozza N , Castelli R , et al. Expanding the arsenal of FGFR inhibitors: a novel chloroacetamide derivative as a new irreversible agent with anti‐proliferative activity against FGFR1‐amplified lung cancer cell lines. Front Oncol. 2019;9:179. doi:10.3389/fonc.2019.00179 30972293 PMC6443895

[42] Wong A , Lamothe B , Lee A , Schlessinger J , Lax I . FRS2 alpha attenuates FGF receptor signaling by Grb2‐mediated recruitment of the ubiquitin ligase Cbl. Proc Natl Acad Sci USA. 2002;99(10):6684‐6689. doi:10.1073/pnas.052138899 11997436 PMC124463

[43] Raffioni S , Thomas D , Foehr ED , Thompson LM , Bradshaw RA . Comparison of the intracellular signaling responses by three chimeric fibroblast growth factor receptors in PC12 cells. Proc Natl Acad Sci USA. 1999;96(13):7178‐7183. 10377388 10.1073/pnas.96.13.7178 PMC22045

[44] Santhana Kumar K , Brunner C , Schuster M , et al. Discovery of a small molecule ligand of FRS2 that inhibits invasion and tumor growth. Cell Oncol. 2023;46(2):331‐356. doi:10.1007/s13402-022-00753-x PMC10060354 36495366

[45] Kähkönen TE , Toriseva M , Petruk N , et al. Effects of FGFR inhibitors TKI258, BGJ398 and AZD4547 on breast cancer cells in 2D, 3D and tissue explant cultures. Cell Oncol. 2021;44(1):205‐218. doi:10.1007/s13402-020-00562-0 PMC7907049 33119860

[46] Härmä V , Virtanen J , Mäkelä R , et al. A comprehensive panel of three‐dimensional models for studies of prostate cancer growth, invasion and drug responses. PLoS One. 2010;5(5):e10431. doi:10.1371/journal.pone.0010431 20454659 PMC2862707

[47] Åkerfelt M , Toriseva M , Nees M . Quantitative phenotypic image analysis of three‐dimensional organotypic cultures. Methods Mol Biol. 2017;1612:433‐445. doi:10.1007/978-1-4939-7021-6_31 28634961

[48] Siciliano T , Simons IH , Beier AMK , et al. A systematic comparison of antiandrogens identifies androgen receptor protein stability as an indicator for treatment response. Lifestyles. 2021;11(9):874. doi:10.3390/life11090874 PMC8468615 34575023

[49] Roskoski R . The role of fibroblast growth factor receptor (FGFR) protein‐tyrosine kinase inhibitors in the treatment of cancers including those of the urinary bladder. Pharmacol Res. 2020;151:104567. doi:10.1016/j.phrs.2019.104567 31770593

[50] Liow E , Howard N , Jung CH , et al. Phase 2 study of neoadjuvant FGFR inhibition and androgen deprivation therapy prior to prostatectomy. Clin Genitourin Cancer. 2022;20(5):452‐458. doi:10.1016/j.clgc.2022.05.007 35688680

[51] Zheng J , Zhang W , Li L , et al. Signaling pathway and small‐molecule drug discovery of FGFR: a comprehensive review. Front Chem. 2022;10:860985. doi:10.3389/fchem.2022.860985 35494629 PMC9046545

[52] Van Bokhoven A , Varella‐Garcia M , Korch C , et al. Molecular characterization of human prostate carcinoma cell lines. Prostate. 2003;57(3):205‐225. doi:10.1002/pros.10290 14518029

[53] Wang L , Huang H , Dougherty G , Zhao Y , Hossain A , Kocher JPA . Epidaurus: aggregation and integration analysis of prostate cancer epigenome. Nucleic Acids Res. 2015;43(2):e7. doi:10.1093/nar/gku1079 25378314 PMC4333365

[54] Mukherjee B , Mayer D . Dihydrotestosterone interacts with EGFR/MAPK signalling and modulates EGFR levels in androgen receptor‐positive LNCaP prostate cancer cells. Int J Oncol. 2008;33(3):623‐629. doi:10.3892/ijo_00000048 18695894

[55] Pignon JC , Koopmansch B , Nolens G , Delacroix L , Waltregny D , Winkler R . Androgen receptor controls EGFR and ERBB2 gene expression at different levels in prostate cancer cell lines. Cancer Res. 2009;69(7):2941‐2949. doi:10.1158/0008-5472.CAN-08-3760 19318561

[56] Giannoni E , Bianchini F , Masieri L , et al. Reciprocal activation of prostate cancer cells and cancer‐associated fibroblasts stimulates epithelial‐mesenchymal transition and cancer stemness. Cancer Res. 2010;70(17):6945‐6956. doi:10.1158/0008-5472.CAN-10-0785 20699369

[57] Straussman R , Morikawa T , Shee K , et al. Tumour micro‐environment elicits innate resistance to RAF inhibitors through HGF secretion. Nature. 2012;487(7408):500‐504. doi:10.1038/nature11183 22763439 PMC3711467

[58] Hirata E , Girotti MR , Viros A , et al. Intravital imaging reveals how BRAF inhibition generates drug‐tolerant microenvironments with high integrin β1/FAK signaling. Cancer Cell. 2015;27(4):574‐588. doi:10.1016/j.ccell.2015.03.008 25873177 PMC4402404

[59] Gotoh N . Regulation of growth factor signaling by FRS2 family docking/scaffold adaptor proteins. Cancer Sci. 2008;99:1319‐1325. doi:10.1111/j.1349-7006.2008.00840.x 18452557 PMC11159094

[60] Labrecque MP , Brown LG , Coleman IM , et al. Targeting the fibroblast growth factor pathway in molecular subtypes of castration‐resistant prostate cancer. Prostate. 2024;84(1):100‐110. doi:10.1002/pros.24630 37796107 PMC10851871

[61] Linxweiler J , Hajili T , Körbel C , et al. Cancer‐associated fibroblasts stimulate primary tumor growth and metastatic spread in an orthotopic prostate cancer xenograft model. Sci Rep. 2020;10(1):12575. doi:10.1038/s41598-020-69424-x 32724081 PMC7387494

[62] Bai Y , Yue C , Lu Z , Li P , Liu H . The role of α‐smooth muscle actin in confirming the microinvasion of laryngeal squamous cell carcinoma. Ann Diagn Pathol. 2021;54:151804. doi:10.1016/j.anndiagpath.2021.151804 34419855

[63] Öhlund D , Handly‐Santana A , Biffi G , et al. Distinct populations of inflammatory fibroblasts and myofibroblasts in pancreatic cancer. J Exp Med. 2017;214(3):579‐596. doi:10.1084/jem.20162024 28232471 PMC5339682

[64] Sahai E , Astsaturov I , Cukierman E , et al. A framework for advancing our understanding of cancer‐associated fibroblasts. Nat Rev Cancer. 2020;20(3):174‐186. doi:10.1038/s41568-019-0238-1 31980749 PMC7046529

[65] Wu SZ , al‐Eryani G , Roden DL , et al. A single‐cell and spatially resolved atlas of human breast cancers. Nat Genet. 2021;53(9):1334‐1347. doi:10.1038/s41588-021-00911-1 34493872 PMC9044823

[66] Pal B , Chen Y , Vaillant F , et al. A single‐cell RNA expression atlas of normal, preneoplastic and tumorigenic states in the human breast. EMBO J. 2021;40(11):e107333. doi:10.15252/embj.2020107333 33950524 PMC8167363

