# Supplementary material for: Targeting the cancer cells and cancer‐associated fibroblasts with next‐generation FGFR inhibitors in prostate cancer co‐culture models
Source: Cancer Med. 2024 Sep 20;13(18):e70240. doi: 10.1002/cam4.70240 (PMC11413502; doi:10.1002/cam4.70240)
Supplement: Supplementary file 1 — Figures S1–S10. [file CAM4-13-e70240-s001.pdf]

## Supplementary Figures

**Targeting the cancer cells and cancer-associated fibroblasts with next-generation FGFR inhibitors in prostate cancer co-culture models.**

Syeda Afshan[1], Yu Gang Kim[1], Jesse Mattsson[1], Malin Åkerfelt[1,2], Pirkko Härkönen[1], Martin Baumgartner[3], Matthias Nees[1,4]

[1] FICAN West Cancer Centre, Institute of Biomedicine, University of Turku, Turku, Finland.
[2]Cell Biology, Faculty of Science and Engineering, Åbo Akademi University, Turku, Finland.
[3]Pediatric Molecular Neuro-Oncology Research Laboratory, University Children's Hospital Zurich, Zurich, Switzerland.
[4]Department of Biochemistry and Molecular Biology, Medical University of Lublin, Lublin, Poland.

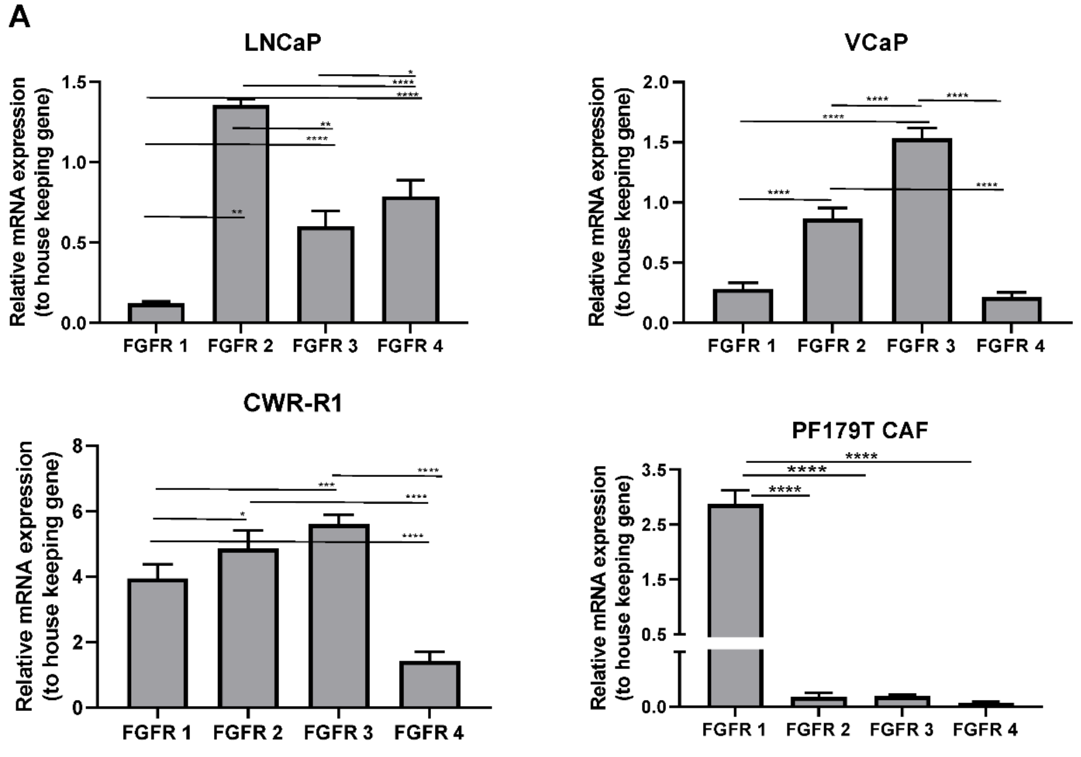


**Supplementary Figure 1. (A)** The mRNA expression of fibroblast growth factor receptors FGFR 1–4 in prostate cancer cell lines LNCaP, VCaP, CWR-R1, and PF179T CAF, as analyzed by qRT-PCR. The boxes indicate the relative mRNA expression levels compared to the housekeeping gene, TATA box binding protein (TBP). The one-way ANOVA with Dunnett's test indicates the statistical significance of differential expression with n=3 replicates. * $p < 0.05$, **$p < 0.01$, *** $p < 0.001$, ****$p < 0.0001$.

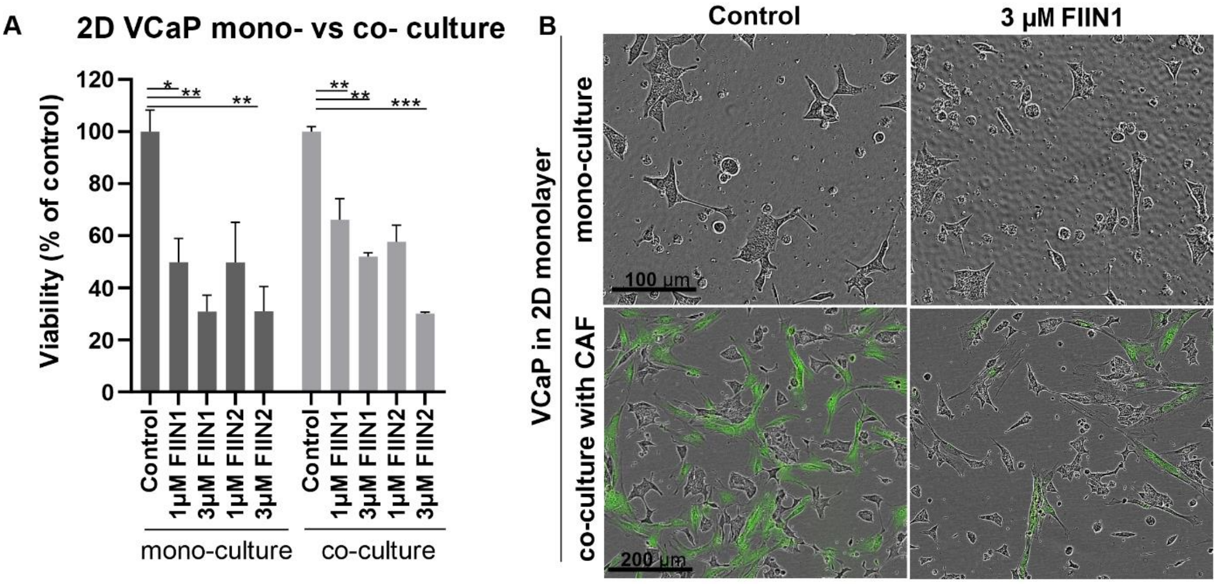


**Supplementary Figure 2.** Reduction of cell viability (in percentage, compared to control) and morphometric effects as a result of FGFRi (FIIN1, FIIN2) treatment in adherent 2D mono-versus co-culture with or without CAFs (green fluorescence). The treatment was done for 72 h in n=3 replicas. **(A)** The relative viability of VCaP cells after treatment with FGFRi in 2D monoculture and co-culture with CAFs compared to control, as measured by Cell Titre Glo metabolic assay. **(B)** Representative IncuCyte phase-contrast images of VCaP 2D cultures with or without CAFs. Scale bars as shown in the images.

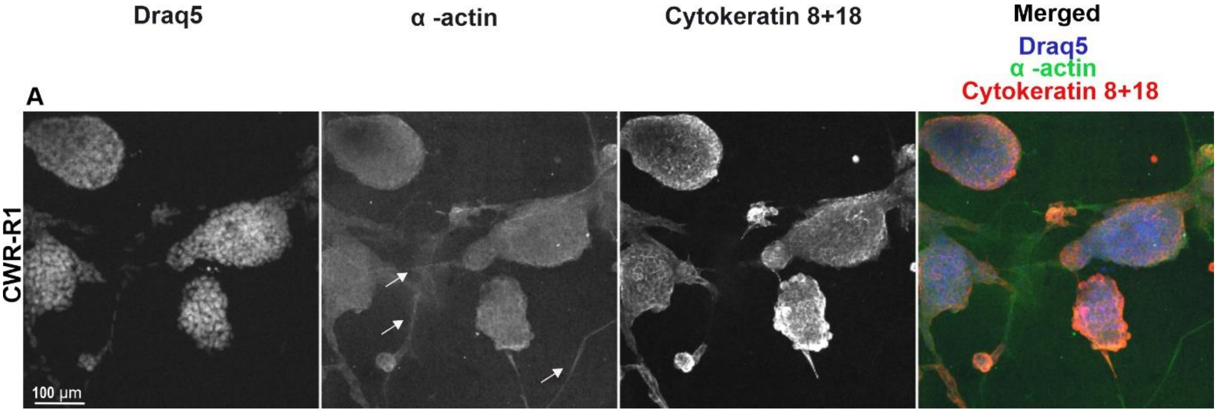


**Supplementary Figure 3.** (**A**) Organotypic 3D co-cultures of CWR-R1 cells in Matrigel and collagen type 1 matrix cultured for 10 days. The organoids and CAFs were counter-stained with Draq5 nuclear stain (blue). Antibodies used for IF staining were α-smooth muscle actin (α-actin, green) as a marker of cancer-associated fibroblasts; indicated with arrows, and epithelial cell marker cytokeratin 8+18 (red). Grayscale images show each marker separately. The images for all three channels were acquired using a spinning disk confocal microscope and merged to generate a composite color image showing the interactions of CWR-R1(red) with its fibroblasts (green) under 20x objective, scale bar=100μm.

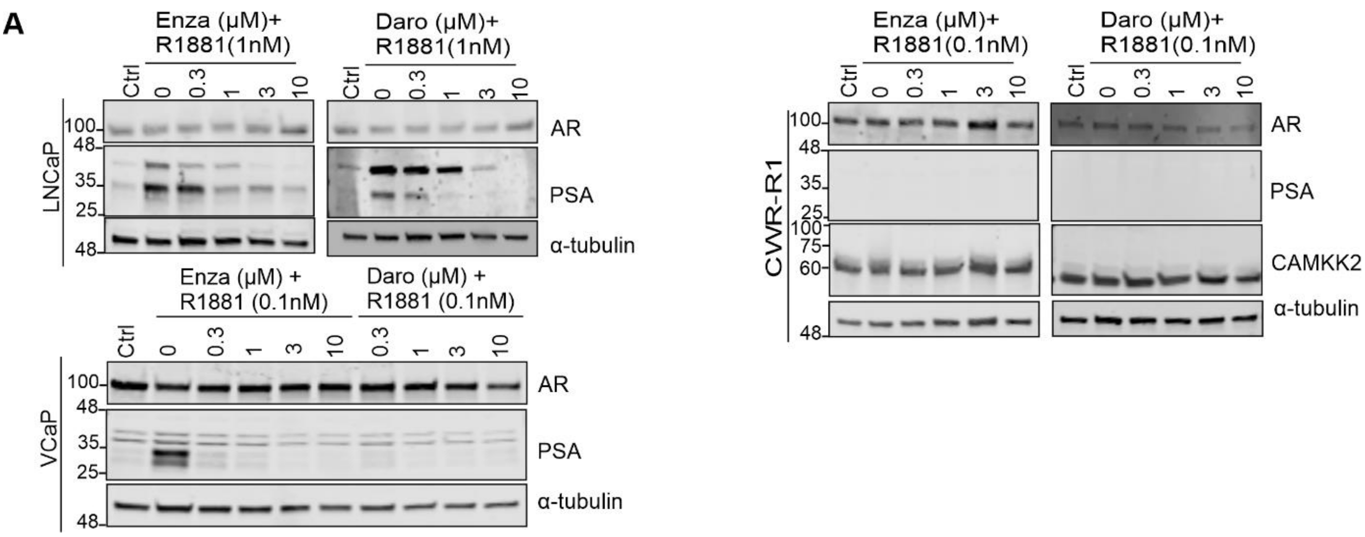


**Supplementary Figure 4.** The effects of AR inhibitors on AR signalling of PCa cells. **(A)** Immunoblot analysis of AR, PSA and CAMKK2 protein expression in LNCaP, CWR-R1 and VCaP cells cultured in charcoal-stripped serum and treated with the synthetic androgen, R1881 for 72 h in the presence or absence of enzalutamide (Enza) and darolutamide (Daro) at the indicated concentrations. α-tubulin was used as a loading control.

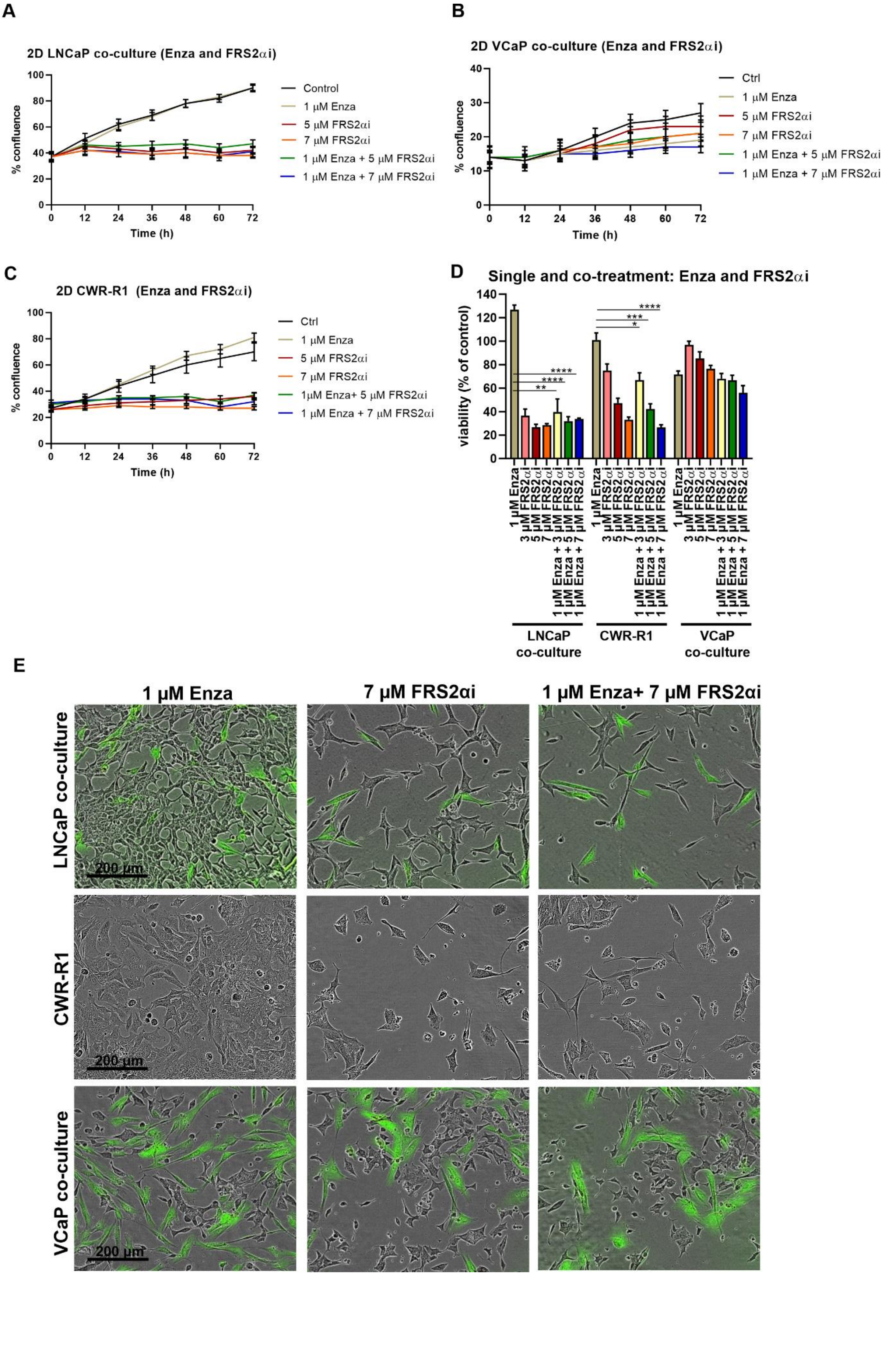
A
2D LNCaP co-culture (Enza and FRS2αi)
% confluence
Time (h)
Control
1 μM Enza
5 μM FRS2αi
7 μM FRS2αi
1 μM Enza + 5 μM FRS2αi
1 μM Enza + 7 μM FRS2αi
B
2D VCaP co-culture (Enza and FRS2αi)
% confluence
Time (h)
Ctrl
1 μM Enza
5 μM FRS2αi
7 μM FRS2αi
1 μM Enza + 5 μM FRS2αi
1 μM Enza + 7 μM FRS2αi
C
2D CWR-R1 (Enza and FRS2αi)
% confluence
Time (h)
Ctrl
1 μM Enza
5 μM FRS2αi
7 μM FRS2αi
1μM Enza+ 5 μM FRS2αi
1 μM Enza + 7 μM FRS2αi
D
Single and co-treatment: Enza and FRS2αi
viability (% of control)
1 μM Enza
3 μM FRS2αi
5 μM FRS2αi
7 μM FRS2αi
1 μM Enza + 3 μM FRS2αi
1 μM Enza + 5 μM FRS2αi
1 μM Enza + 7 μM FRS2αi
LNCaP co-culture
CWR-R1
VCaP co-culture
E
1 μM Enza
7 μM FRS2αi
1 μM Enza+ 7 μM FRS2αi
LNCaP co-culture
CWR-R1
VCaP co-culture
200 μm

**Supplementary Figure 5.** Effects of FRS2αi compound on PCa cell lines LNCAP, VCAP, and CWR-R1, combined with ARi enzalutamide (Enza). Incucyte real-time cell growth and cellular activity analysis of 2D cell cultures and co-cultures upon treatment is shown. **(A)** Proliferation curves over 72 h are expressed as (percentage %) cell confluency relative to solvent control for LNCAP and CAF co-cultures, and **(B)** for VCAP and CAF co-cultures, with drug concentrations and combinations as indicated. **(C)** Identical treatment is shown for the CWR-R1 cell line, which retains a stable population of mouse fibroblasts. **(D)** Relative viability of LNCAP and VCAP co-cultured with CAFs as well as CWR-R1 cells after exposure to FRS2αi alone or in combination with Enza for 72 h, compared to control (viability=100%). Statistics: one-way ANOVA using Dunnett's test with 1 μM Enza as reference (*$p < 0.05$, ** $p < 0.01$, ***$p < 0.001$, ****$p < 0.0001$). (**E**) Representative phase contrast images of treatment effects in LNCAP, VCAP, and CWR-R1 cells in 2D co-culture with CAFs. Treatments: FRS2αi (3 μM, 5 μM and 7 μM) and enzalutamide (1 μM) alone, or in combination for 72 h .

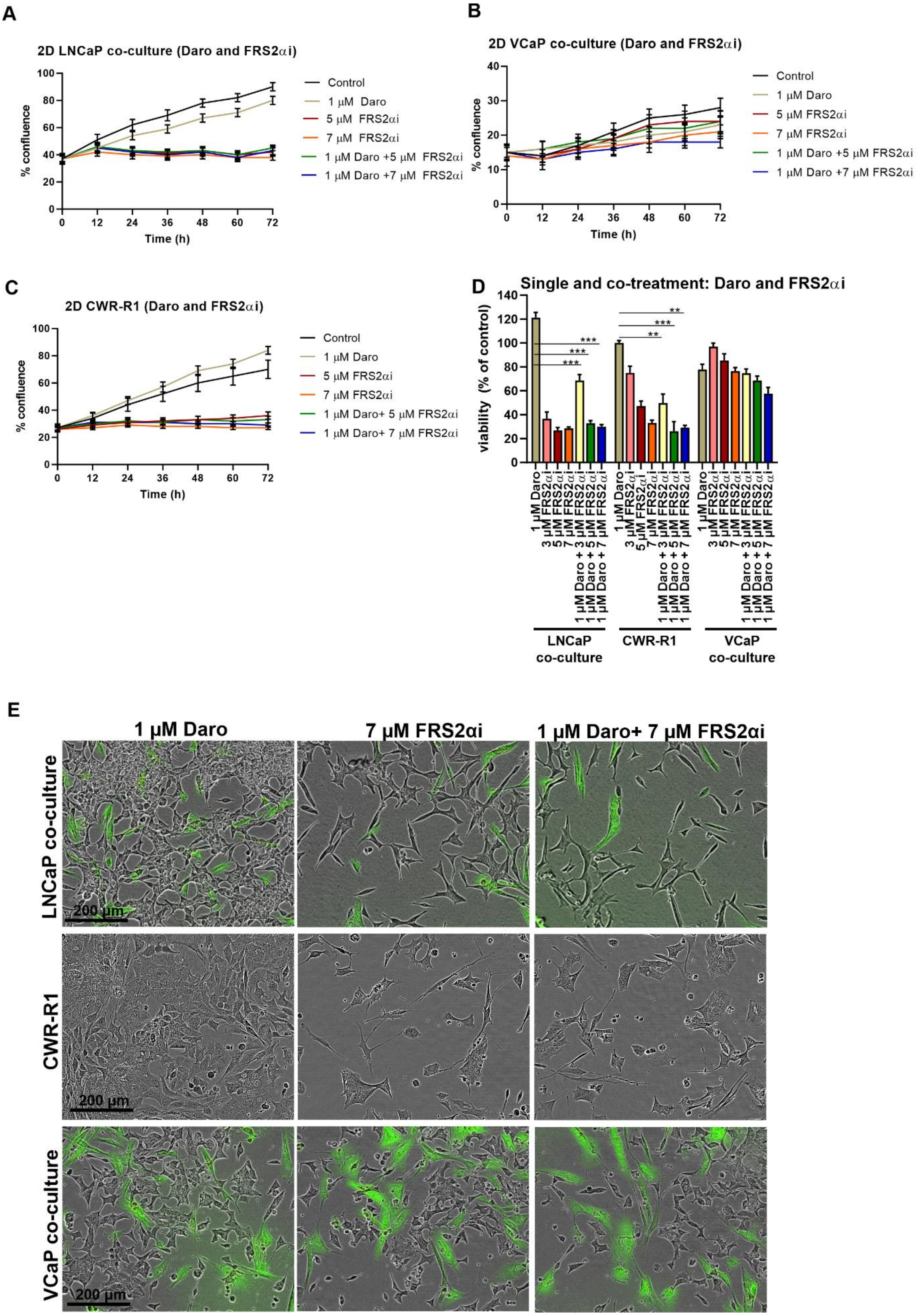

A
2D LNCaP co-culture (Daro and FRS2αi)
% confluence
Time (h)
Control
1 μM Daro
5 μM FRS2αi
7 μM FRS2αi
1 μM Daro +5 μM FRS2αi
1 μM Daro +7 μM FRS2αi
B
2D VCaP co-culture (Daro and FRS2αi)
% confluence
Time (h)
C
2D CWR-R1 (Daro and FRS2αi)
% confluence
Time (h)
D
Single and co-treatment: Daro and FRS2αi
viability (% of control)
1 μM Daro
3 μM FRS2αi
5 μM FRS2αi
7 μM FRS2αi
1 μM Daro + 3 μM FRS2αi
1 μM Daro + 5 μM FRS2αi
1 μM Daro + 7 μM FRS2αi
LNCaP co-culture
CWR-R1
VCaP co-culture
E
1 μM Daro
7 μM FRS2αi
1 μM Daro+ 7 μM FRS2αi
LNCaP co-culture
CWR-R1
VCaP co-culture
200 μm

**Supplementary Figure 6.** Effects of FRS2αi compound on PCa cell lines, as shown in Suppl. Fig 5., but combined with ARi darolutamide (Daro). **(A)** Proliferation curves over 72 h are expressed as (percentage %) cell confluency relative to solvent control for LNCAP and CAF co-cultures, and **(B)** for VCAP and CAF co-cultures. **(C)** Identical treatment is shown for the CWR-R1 cell line. **(D)** Relative viability of LNCAP and VCAP co-cultured with CAFs and CWR-R1 cells after exposure to FRS2αi alone or in combination with Daro for 72 h, compared to control (viability=100%). Statistics: one-way ANOVA using Dunnett's test with 1 μM Daro as reference (*p < 0.05, ** p < 0.01, ***p < 0.001, ****p < 0.0001). **(E)** Representative phase contrast images of treatment effects in LNCAP, VCAP, and CWR-R1 cells in 2D co-culture with CAFs. Treatments: FRS2αi (3 μM, 5 μM and 7 μM) and darolutamide (1 μM) alone, or in combination for 72 h.

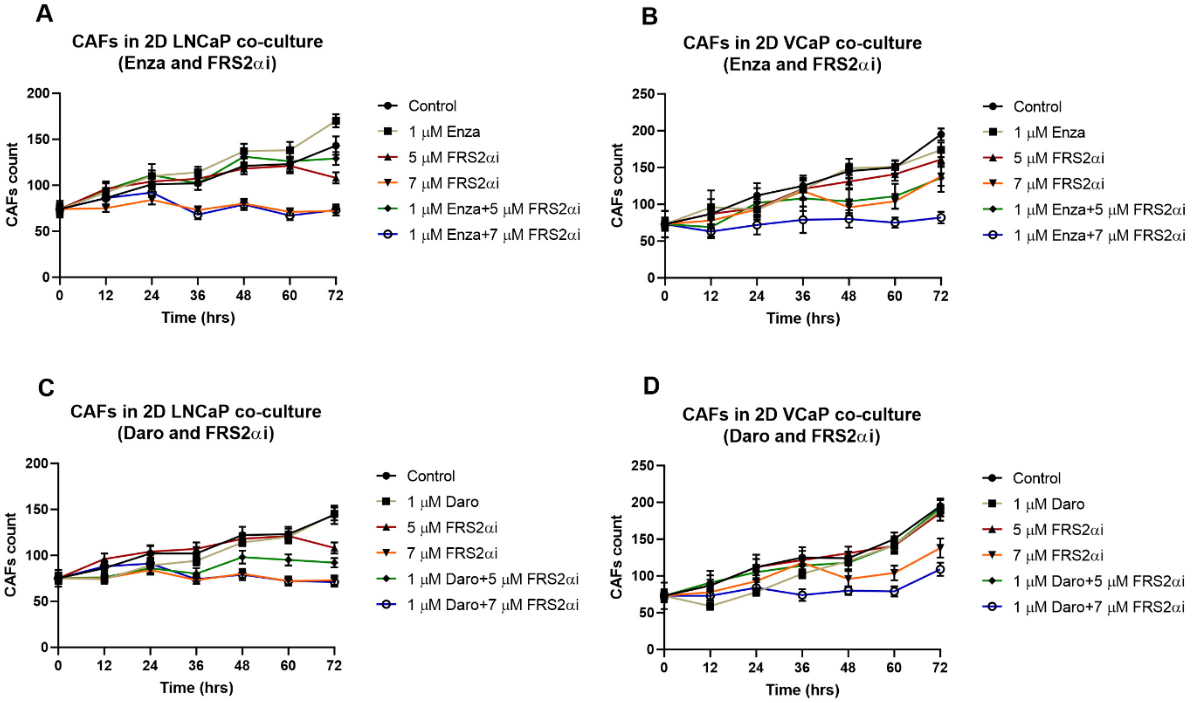


**Supplementary Figure 7.** The concentration-dependent decrease in the average cell count of CAFs (green fluorescence cells) upon single treatment and in combination with FRS2αi and ARi, enzalutamide (Enza) or darolutamide (Daro) as depicted in the graphs for 72 h in **(A, C)** 2D LNCaP co-culture and **(B, D)** 2D VCaP co-culture.

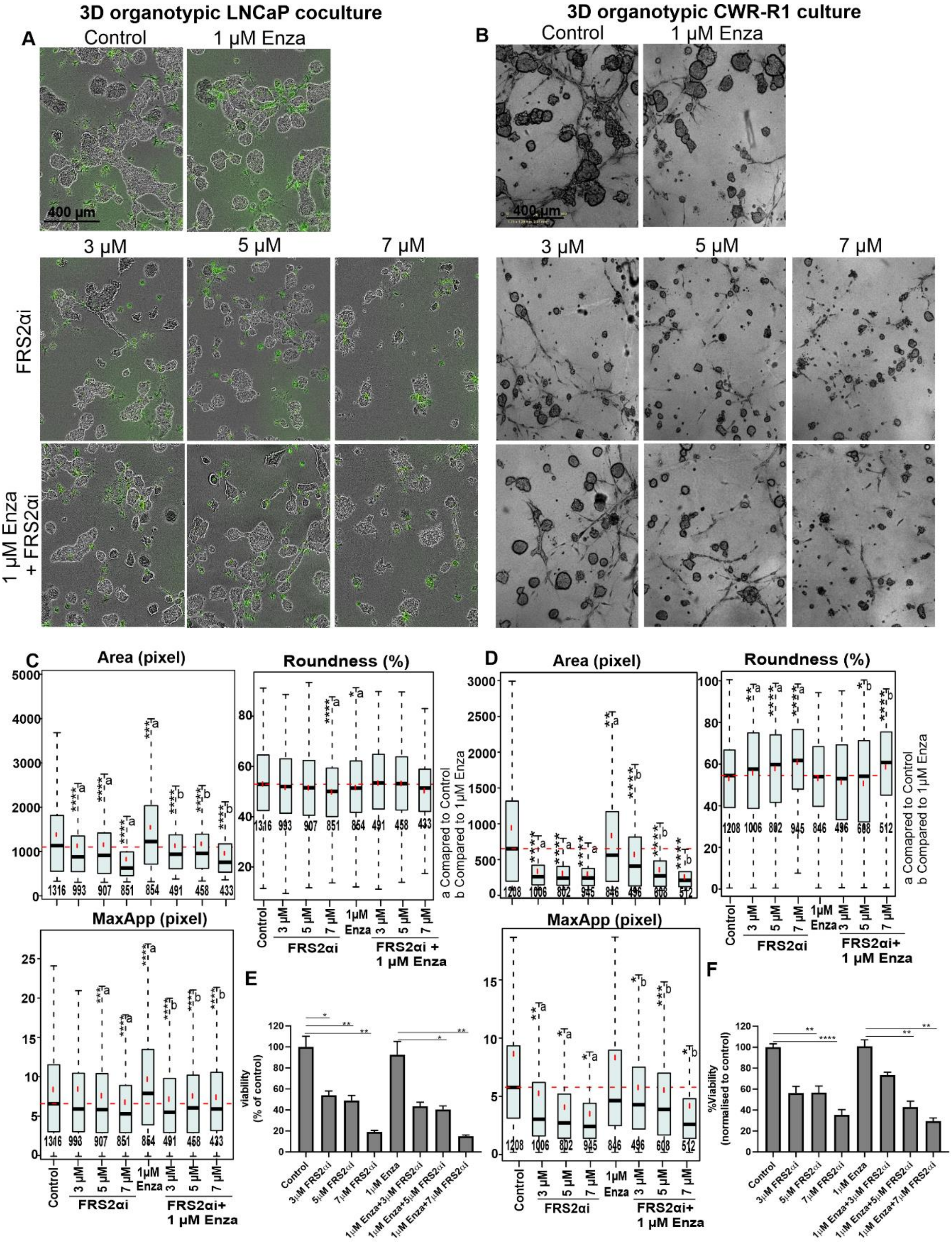


**Supplementary Figure 8.** Compound FRS2αi represses growth of PCa cells co-cultured with CAFs in our 3D-organotypic model, but shows no synergistic or added efficacy when combined with Enzalutamide (Enza). **(A)** Representative phase contrast and green fluorescence images of organotypic cultures formed by LNCAP+CAF. (**B)** Brightfield microscope images of CWR-R1 organotypic cultures at the end point of treatment with FRS2αi alone or in combination with 1μM Enza. Scale bar 400 μm. Quantitative phenotypic analysis (**C**) of 3D LNCaP and (**D)** CWR-R1co-cultures. AMIDA image analysis software was used to measure organoid size in

pixels (Area), roundness of organoids (in percentage) and the maximum size of appendages (MaxApp in pixel). Box and whisker plots represent medians of treated sample (black horizontal line), median of control sample (dotted red horizontal line) and the number of objects in the analyses. Statistical significances of n = 3 replica calculated using Bonferroni-corrected t-test (*p < 0.05, ** p < 0.01, ***p < 0.001, ****p < 0.0001). Viability analysis of 3D **(E)** LNCaP co-culture and **(F)** CWR-R1, exposed to FRS2αi alone or combined with 1 µM of Enza. One-way ANOVA using Dunnett's test of n = 3 replicas (*p < 0.05, ** p < 0.01, ***p < 0.001, ****p < 0.0001).

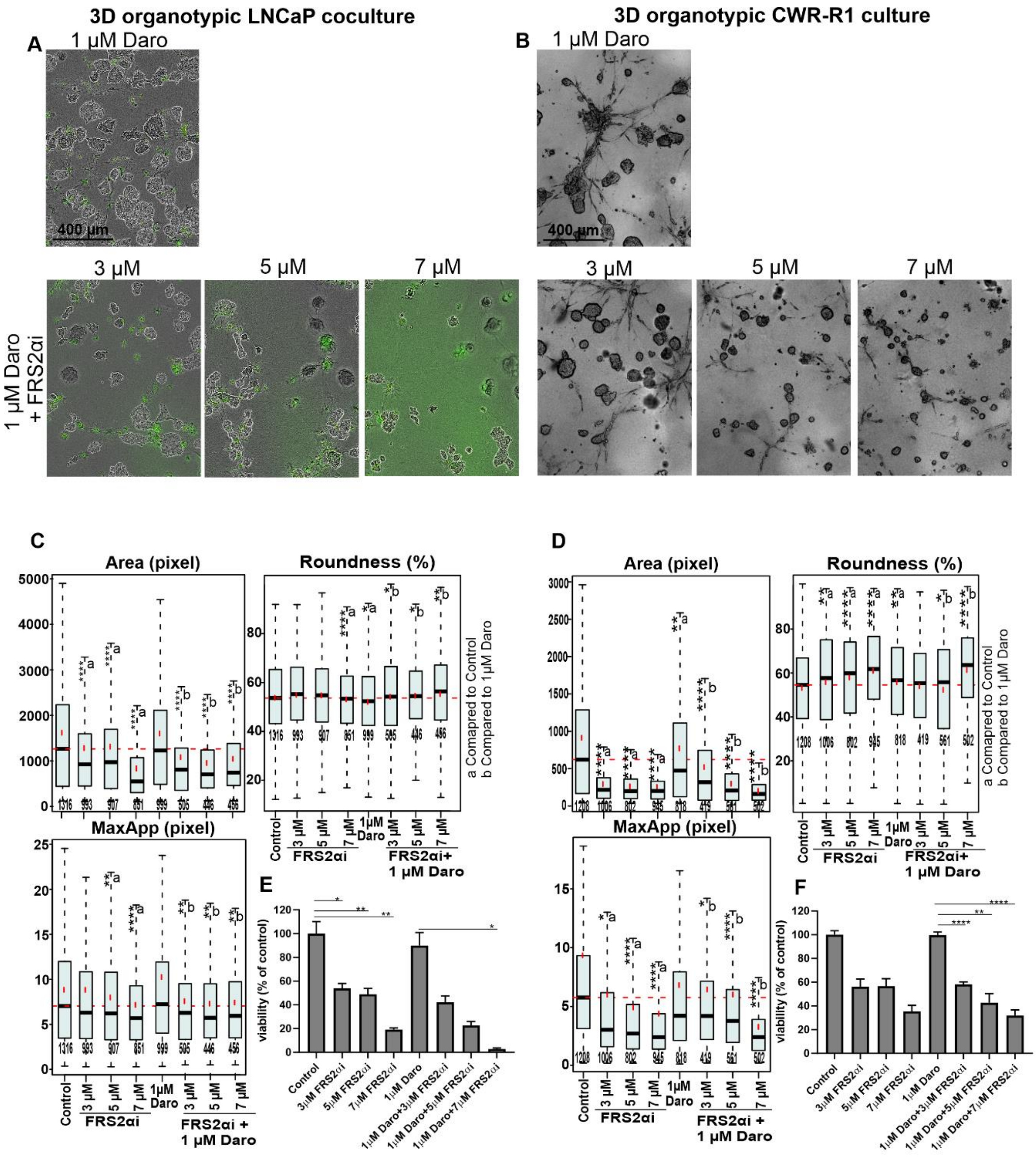


**Supplementary Figure 9.** FRS2αi represses PCa growth in 3D-organotypic co-cultures with CAFs and shows no synergistic or added efficacy when combined with Darolutamide (Daro). **(A)** Representative phase contrast images of LNCAP and GFP-tagged CAFs in 3D co-cultures.

(**B)** Brightfield microscope images of CWR-R1 at the end point of treatment with FRS2αi alone or in combination with 1μM Daro. Scale bar of 400 μm. Quantitative phenotypic analysis of 3D LNCaP organoids (**C)** and CWR-R1 (**D).** Organotypic structure area in pixel (Area), the roundness of organoids (in percentage), and the maximum size of appendages (MaxApp in pixel) were measured. Box and whisker plots represent the medians of the treated sample (black horizontal line), the median of the control sample (dotted red horizontal line), and the number of objects in the analyses. Statistical significances of n = 3 replicates calculated using Bonferroni-corrected t-test (*$p < 0.05$, ** $p < 0.01$, ***$p < 0.001$, ****$p < 0.0001$). (**E, F**) Viability analysis of LNCaP 3D co-culture indicated in **E** and CWR-R1 in **F**, exposed to FRS2αi alone or in combination with 1 μM of Daro. One-way ANOVA using Dunnett's test of n = 3 replicas (*$p < 0.05$, ** $p < 0.01$, ***$p < 0.001$, ****$p < 0.0001$). The untreated, vehicle (0.2% DMSO) control and images of single treatment of FRS2αi can be referred to in Supplementary Figure 8.

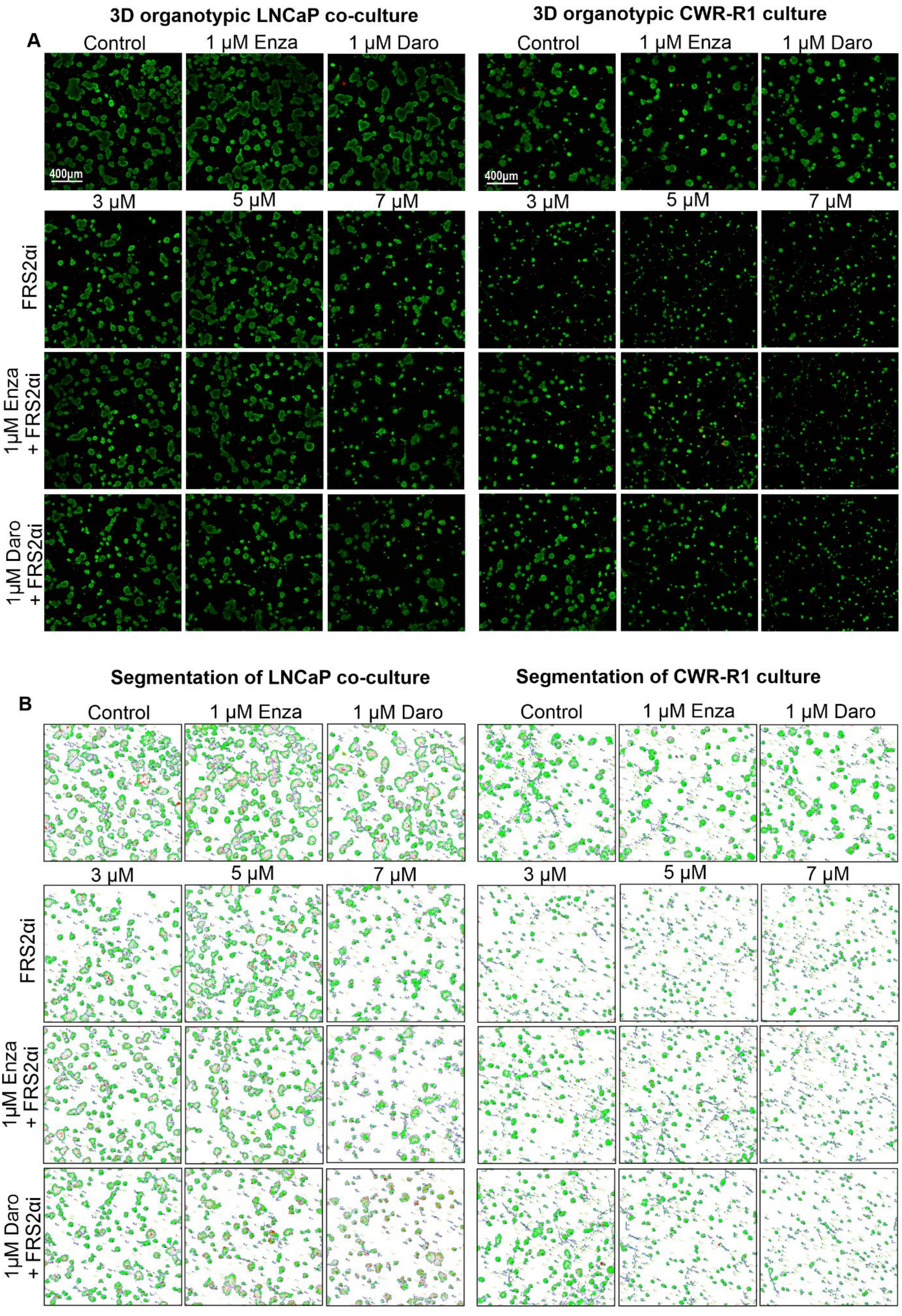

3D organotypic LNCaP co-culture
3D organotypic CWR-R1 culture
A
Control
1 µM Enza
1 µM Daro
400µm
3 µM
5 µM
7 µM
FRS2αi
1µM Enza + FRS2αi
1µM Daro + FRS2αi
Segmentation of LNCaP co-culture
Segmentation of CWR-R1 culture
B

**Supplementary Figure 10:** Confocal imaging of 3D organotypic cultures using a spinning disk microscope followed by phenotypic image analysis using AMIDA software**. (A)** The organotypic cultures were stained with live cell stain Calcein-AM (green) after 6 days of inhibitor treatments. 5x objective was used to capture these representative images. **(B)** Segmentation of the maximum projection images was done using the AMIDA software. Automated quantitation for various phenotypic parameters of the 3D structures was performed as described in Figure 5 and in Supplementary Figs. 8 and 9.
