# Supplementary material for: Targeting the cancer cells and cancer‐associated fibroblasts with next‐generation FGFR inhibitors in prostate cancer co‐culture models
Source: Cancer Med. 2024 Sep 20;13(18):e70240. doi: 10.1002/cam4.70240 (PMC11413502; doi:10.1002/cam4.70240)
Supplement: Supplementary file 2 — Table S1. [file CAM4-13-e70240-s002.docx]

**Targeting the cancer cells and cancer-associated fibroblasts with next-generation**

**FGFR inhibitors in prostate cancer co-culture models.**

Syeda Afshan^1^, Yu Gang Kim^1^, Jesse Mattsson^1^, Malin Åkerfelt^1,2^, Pirkko Härkönen^1^, Martin Baumgartner^3^, Matthias Nees^1,4^

^1^ FICAN West Cancer Centre, Institute of Biomedicine, University of Turku, Turku, Finland.

^2^Cell Biology, Faculty of Science and Engineering, Åbo Akademi University, Turku, Finland.

^3^Pediatric Molecular Neuro-Oncology Research Laboratory, University Children’s Hospital Zurich, Zurich, Switzerland.

^4^Department of Biochemistry and Molecular Biology, Medical University of Lublin, Lublin, Poland.

| **Inhibitor** |  |  | **IC50(nM)** |  |  | **Reference** |
| --- | --- | --- | --- | --- | --- | --- |
|  | FGFR1 | FGFR2 | FGFR3 | FGFR4 | Other targets |  |
| FIIN1 | 9.2 | 6.2 | 11.9 | 189 | Flt1/VEGFR (661nM), Blk (381nM) | ^1^ |
| FIIN2 | 3.1 | 4.3 | 27 | 45 | EGFR (204nM) | ^2^ |
| FRS2αi  (experimental compound 7) | NA | NA | NA | NA | NA. IC50 is less than 20µM in various cancer types as stated in the reference article. | ^3^ |

**Supplementary Table 1:** The IC50 values of the next-generation FGFR inhibitors used in this study. NA refers to no binding to the receptor or protein.

1. Zhou, W. *et al.* A Structure-Guided Approach to Creating Covalent FGFR Inhibitors. *Chem Biol* **17**, 285–295 (2010).

2. Tan, L. *et al.* Development of covalent inhibitors that can overcome resistance to first-generation FGFR kinase inhibitors. *Proc Natl Acad Sci U S A* **111**, E4869–E4877 (2014).

3. Santhana Kumar, K. *et al.* Discovery of a small molecule ligand of FRS2 that inhibits invasion and tumor growth. *Cellular Oncology* **46**, 331–356 (2023).
